# A Novel Mouse Home Cage Lickometer System Reveals Sex- and Housing-Based Influences on Alcohol Drinking

**DOI:** 10.1523/ENEURO.0234-24.2024

**Published:** 2024-10-04

**Authors:** Nicholas Petersen, Danielle N. Adank, Yizhen Quan, Caitlyn M. Edwards, Sabrina D. Hallal, Anne Taylor, Danny G. Winder, Marie A. Doyle

**Affiliations:** ^1^Vanderbilt Brain Institute, Vanderbilt University School of Medicine, Nashville, Tennessee 37232; ^2^Vanderbilt Center for Addiction Research, Vanderbilt University School of Medicine, Nashville, Tennessee 37232; ^3^Department of Molecular Physiology and Biophysics, Vanderbilt University School of Medicine, Nashville, Tennessee 37232; ^4^Department of Neurobiology, UMass Chan Medical School, Worcester, Massachusetts 01655

**Keywords:** alcohol, microstructure, open-source, sex differences, social drinking, social isolation

## Abstract

Alcohol use disorder (AUD) is a significant global health issue. Despite historically higher rates among men, AUD prevalence and negative alcohol-related outcomes in women are rising. Loneliness in humans has been associated with increased alcohol use, and traditional rodent drinking models involve single housing, presenting challenges for studying social enrichment. We developed LIQ PARTI (Lick Instance Quantifier with Poly-Animal RFID Tracking Integration), an open-source tool to examine home cage continuous access two-bottle choice drinking behavior in a group-housed setting, investigating the influence of sex and social isolation on ethanol consumption and bout microstructure in C57Bl/6J mice. LIQ PARTI, based on our previously developed single-housed LIQ HD system, accurately tracks drinking behavior using capacitive-based sensors and RFID technology. Group-housed female mice exhibited higher ethanol preference than males, while males displayed a unique undulating pattern of ethanol preference linked to cage changes, suggesting a potential stress or novelty-related response. Chronic ethanol intake distinctly altered bout microstructure between male and female mice, highlighting sex and social environmental influences on drinking behavior. Social isolation with the LIQ HD system amplified fluid intake and ethanol preference in both sexes, accompanied by sex- and fluid-dependent changes in bout microstructure. However, these effects largely reversed upon resocialization, indicating the plasticity of these behaviors in response to social context. Utilizing a novel group-housed home cage lickometer device, our findings illustrate the critical interplay of sex and housing conditions in voluntary alcohol drinking behaviors in C57Bl/6J mice, facilitating nuanced insights into the potential contributions to AUD etiology.

## Significance Statement

Loneliness has been associated with increased alcohol use, and traditional rodent drinking models involve single housing, presenting challenges for studying social enrichment. Here we developed LIQ PARTI (Lick Instance Quantifier with Poly-Animal RFID Tracking Integration), an open-source group-housed lickometer system to investigate how social housing, isolation, and sex influence alcohol consumption patterns in C57Bl/6J mice. LIQ PARTI accurately identifies mouse drinking events and uncovered significant housing- and fluid-dependent differences in ethanol consumption and bout microstructure between male and female mice. Social isolation-induced changes to ethanol drinking behavior and microstructure were highly plastic, as resocialization generally reversed these changes. These findings expand on the complex interplay between sex, social isolation, and alcohol use.

## Introduction

Alcohol use disorder (AUD) is highly prevalent in the United States and worldwide (Grant et al., 2015). There are significant sex differences in alcohol use, with a lifetime AUD prevalence of 36% in men, compared with 22.7% in women (Grant et al., 2015; Flores-Bonilla and Richardson, 2020). However, the gap is narrowing due to an increase in alcohol use in women (White, 2020). Additionally, alcohol-related negative outcomes have increased overall for US adults but have increased more in women compared with men (White, 2020). In rodents, female mice and rats tend to exhibit increased ethanol consumption compared with males, especially in the commonly used C57Bl/6J mouse strain (Middaugh et al., 1999; Lopez and Laber, 2015; Centanni et al., 2019; Sneddon et al., 2019; Yu et al., 2019; Flores-Bonilla and Richardson, 2020; M. A. Caruso et al., 2021; Rivera-Irizarry et al., 2023).

Social isolation has significant impacts on behavior and neurophysiology, including the effects of alcohol and alcohol consumption (Butler et al., 2016). Prolonged social isolation potentiates the locomotor-inducing effects of ethanol injection in mice (Päivärinta, 1990), and studies utilizing various models of ethanol consumption report that social isolation prior to ethanol exposure increases later ethanol consumption (Lopez et al., 2011; Sanna et al., 2011; Butler et al., 2014a; Lopez and Laber, 2015; Cortés-Patiño et al., 2016; Panksepp et al., 2017; Evans et al., 2020; Cullins and Chester, 2024). Given that most rodent drinking models require animals to be singly housed for accurate individual intake measurements, the effects of sex and social environments on continuous access ethanol consumption, preference, and drinking patterns in ethanol-experienced mice are currently poorly understood.

The assessment of drinking in group-housed rodents has previously required the use of drinking models where ethanol consumption is recorded in discrete, limited sessions where animals are singly housed (Lopez et al., 2011; Sanna et al., 2011; Butler et al., 2014a,b; Lopez and Laber, 2015; Cortés-Patiño et al., 2016; Cullins and Chester, 2024), separated by a physical barrier (McKenzie-Quirk and Miczek, 2008; Evans et al., 2020), or recorded as an average of all animals in a cage (Panksepp et al., 2017). Specialized housing environments, such as the IntelliCage (Holgate et al., 2017) and HM2 cage (Fulenwider et al., 2021), have been valuable tools in studying the effects of social housing and enrichment on consummatory behavior. However, these systems are costly and require specialized housing. Tracking multiple animals in a cage can be performed with implanted radiofrequency identification (RFID) tags, which have been utilized in commercially available systems and by recently developed open-source behavioral devices to identify group-housed mice with video-assisted tracking in an arena (Peleh et al., 2019), mice in specialized housing (Wong et al., 2023), and rats during home cage drinking (Frie and Khokhar, 2024).

We previously developed the open-source tool LIQ HD (Lick Instance Quantifier Home cage Device; Petersen et al., 2023), a home cage lickometer system that utilizes capacitive sensor-based technology to record undisturbed two-bottle choice mouse drinking behavior and lick microstructure in individually housed mice. Here we present LIQ PARTI (Lick Instance Quantifier with Poly-Animal RFID Tracking Integration), an open-source system designed and built based on the LIQ HD technology with the addition of RFID tags and readers for group-housed mice. Unlike currently available systems for mice, LIQ PARTI is compatible with normal vivarium home cages and can be modified to fit other cage models. Like LIQ HD, this system accurately tracks licking behavior and bout microstructure undisturbed by experimenters over at least a week, is inexpensive, and is easy to assemble with the use of 3D-printed parts and commercially available electronic components.

Using LIQ PARTI and LIQ HD, we investigated the role of sex and social environments on continuous access two-bottle choice ethanol consumption and bout microstructure in male and female C57Bl/6J mice. We found that male and female mice display significant differences in ethanol preference, drinking patterns, and drink bout microstructure in a group-housed environment that differ from those observed in an isolated setting. Group-housed females have a significantly higher preference for ethanol, while males display an undulating pattern of ethanol preference that aligns with cage bedding changes. Both male and female mice increase their ethanol preference and total consumption when transitioned into the socially isolated LIQ HD system and show significant sex- and bottle-dependent changes to bout microstructure. Drinking changes due to social isolation generally normalize following reintroduction to a group-housed environment. Altogether, we identified sex- and malleable housing-based influences on alcohol drinking using a novel open-source group-housed drinking device.

## Materials and Methods

### Animals

Male and female C57BL/6J mice (8 weeks of age) were purchased from Jackson Laboratory (#000664). Mice were allowed to habituate to the animal facility for at least 7 d before the start of experimentation. All mice were housed in Lab Products Super Mouse 750 Ventilated Cages with Diamond Soft paper bedding on a standard 12 h light/dark cycle at 22–25°C with food (LabDiet 5LOD) and water available *ad libitum*. All bottle weight measurements and cage changes conducted by experimenters occurred during the light phase. All experiments were approved by the Vanderbilt University Institutional Animal Care and Use Committee (IACUC) and were carried out in accordance with the guidelines set in the Guide for the Care and Use of Laboratory Animals of the National Institutes of Health.

### LIQ PARTI build

A detailed LIQ PARTI step-by-step guide, along with the Arduino code and 3D models, can be found at https://github.com/nickpetersen93/LIQ_PARTI. LIQ PARTI is built from commercially available electronic components combined with 3D-printed parts (Extended Data Fig. 1-1*A,B*). Each cage is controlled by a single Arduino MEGA microcontroller connected to a real-time clock plus data logging shield (Adafruit), a capacitive touchscreen shield (Adafruit), a 12-channel MPR121 capacitive sensor breakout board (Adafruit), and two 125 kHz RFID readers (SparkFun). Based on the previously published LIQ HD two-bottle choice system (Petersen et al., 2023), LIQ PARTI utilizes capacitive touch sensing to measure individual lick events in group-housed mice. The metal sipper for each bottle is in contact with conductive copper foil tape that is wired to the MPR121 capacitance sensor breakout board. The two RFID readers lay inside the 3D-printed housing above two small tunnels that the mice must enter to reach each sipper, which allows the device to accurately determine which mouse is actively drinking from each sipper. Both sippers can be used simultaneously without affecting data collection. Date and time are kept with the Adafruit Data Logger Shield, which also writes the cage data to a CSV file on an SD card for downstream analysis. A 2.8″ Adafruit Touchscreen Shield is used to display an easy-to-use graphical user interface, allowing the user to change device settings (time, light/dark schedule, sensor sensitivity, metrics to record), mount/eject the SD card, track the total licks at each sipper throughout recordings, and start/stop/pause recordings. The screen will also prompt the user if there are any errors, such as disconnected sensors or SD card.

The Data Logger Shield and Touchscreen Shield were modified as previously described (Petersen et al., 2023). Briefly, a connection must be made between the “CS” pin to pin 7 on the Data Logger, and the connection on the “CS” solder pad must be cut. On the Touchscreen Shield, a solder bridge across the “back lite #5” pads allows for dimming of the screen during the animals’ dark cycle. Mount the Data Logger Shield and the Touchscreen Shield onto the Arduino Mega by aligning and inserting the headers. The MPR121 breakout board and RFID readers communicate with the Arduino Mega via I^2^C, which allows the microcontroller to communicate with multiple devices connected to the same pins if they have different I^2^C addresses. The MPR121 Board and RFID reader #1 (LEFT side) will remain unmodified, but to modify the I^2^C address of the RFID reader #2 (RIGHT side), create a solder bridge across the “ADR” pads. Additionally, cut the “BUZZER” jumper on both RFID reader boards and mount the ID-12LA RFID reader module onto the boards’ headers. Solder a piece of stranded wire to the “INT” pins on the RFID boards and attach them to the stripped ends of a two-pin connector (for consistency, red wire for the LEFT RFID reader, and black wire for the RIGHT RIFD reader). Next, cut a pair of Male/Male jumper wires (Adafruit) in half and solder the stripped ends of each wire to pins 0 and 1 on the MPR121 (for consistency, red wire for 0, and black wire for 1). Using the other half of the jumper wires, solder each stripped end to a piece of 3″×¼″ conductive copper tape, secure the tape inside each sipper clip, and insert each clip into the device body (red wire for the left side and black wire for the right side). The MPR121 board and RFID readers are connected in series with Qwiic connection cables (SparkFun) and connected to the Qwiic connector on the Touchscreen Shield. Older models of the touchscreen do not have a Qwiic connector, so a Qwiic cable with breadboard jumpers can be connected directly to the Arduino Mega (blue, pin 20; yellow, pin 21; red, 5 V; black, GND), secured with hot glue, and connected with an adapter (SparkFun). A ground wire is connected to the Arduino GND pin and used to ground the system during recordings. Finally, the electronics are secured in the base of the 3D-printed housing.

All 3D models were generated with Shapr3D, and 3D-printed components were printed with PETG filament on an Ultimaker S5 printer as previously described (Petersen et al., 2023). PETG was chosen for its high strength, durability, chemical resistance, ease of use, and food-safe properties. Bottles were printed with translucent filament and then coated internally with food-safe epoxy resin (meets regulation requirements for repeated use under US FDA 21 CFR 177.2600) as previously described (Petersen et al., 2023). The bottles are temperature sensitive and should be washed by hand in warm water with high-quality soap (Dawn Platinum Dish Soap or Alconox Liquinox) or through a Steris Reliance 400XLS Laboratory Glassware Washer using a modified “Plastics” cycle (water temperature <50°C, no heated drying). If sterilization is required, the bottles can be sterilized via gas or UV sterilization methods. The in-cage device body was printed in pieces with black PETG, and the top bottle rack was assembled to the device base with hot glue. The Arduino microcontroller case was printed with a generic PLA material.

The LIQ PARTI Arduino code was uploaded using the open-source Arduino IDE software (version 2.3.2 on MacOS). Users must first install the necessary libraries through the Arduino IDE before uploading the code.

### LIQ PARTI operation (group housed)

The four LIQ PARTI cages were powered by a single 12 V power supply fitted with a four-way DC power splitter. The device operates in a similar manner to LIQ HD, as previously described (Petersen et al., 2023), except that data are logged as individual time-stamped bouts (detailed below) rather than in 1 min bins. Briefly, settings can be changed by pressing the cogwheel icon. The user should designate which side the “experimental” solution is on in the cages (e.g., sucrose, quinine, ethanol, etc.) before pressing “Start” to initiate recording. The capacitive sensor has been further optimized with improved baseline tracking and touch settings for more accurate lick detection. Default settings are preloaded, but users may choose to determine which sensor threshold works best for them. During recordings, users can refresh the lick display page, pause the recording, and save and quit as previously described (Petersen et al., 2023).

### RFID tag implant surgery

Mice were implanted with a 125 kHz Glass Tube Radio Frequency Identification (RFID) Tag (GAO RFID, Product#111001), measuring 2.12 mm in diameter and 12.0 mm in length, subcutaneously on the scruff of the neck (Extended Data Fig. 1-1*C*). The RFID tag is specifically designed for use in animal identification with ISO 11784, ISO 11785, and EM4100 compliance and AMA 1234 and EU3456 regulation specifications. RFID tags were sterilized with a steam autoclave. For the implantation procedure, mice were induced with 3% isoflurane with oxygen and then maintained on 1.5% isoflurane for the remainder of the procedure. Mice were given a single preoperative subcutaneous injection of 2.5 mg/kg meloxicam analgesic. A small (<10 mm) skin incision was made at the implantation site and the skin was carefully released from the underlying muscle using small surgical scissors. The RFID tag was inserted in parallel with the mouse's body with either sterilized forceps or a sterilized injector syringe and the incision was closed with a 6-0 nylon suture. Mice were allowed to recover for 7 d before the start of experimentation.

### Determination of drinking bouts

Unlike the original LIQ HD system (Petersen et al., 2023), LIQ PARTI logs each individual drinking bout rather than logging data in 1 min bins. Thus, only licks that occur in a defined bout are recorded. The start of a drinking bout is defined as three licks in <1 s and the end of a drinking bout is defined as no licks within 1 s. Lick number is defined here as the number of times the animal licked the sipper during a bout, while lick duration is defined as the actual contact time on the sipper. Bout duration is defined as the time from the start of the first lick in a bout to the end of the last lick in a bout. Bout size is defined as the number of licks that occur during each bout. The average lick frequency and interlick interval (ILI) are automatically calculated on the Arduino for each drinking bout. Lick frequency is defined as licks per second during bouts and calculated with the equation:

$$\mathrm{Lick}\;\mathrm{Frequency}\;(\mathrm{Hz})=\frac{\mathrm{Bout}\;\mathrm{Length}\;(\mathrm{licks})}{\mathrm{Bout}\;\mathrm{Duration}\;(\sec)}.$$

The estimated ILI is defined as the time between the offset of a lick and the onset of the subsequent lick and is calculated by subtracting the total bout lick duration from the bout duration and dividing by the total bout length:

$$\mathrm{Estimated}\;\mathrm{Inter}\text{-}\mathrm{Lick}\;\mathrm{Interval}\;(\mathrm{ms})=\frac{\mathrm{Bout}\;\mathrm{Duration}\;(\mathrm{ms})-\;\mathrm{Bout}\;\mathrm{Lick}\;\mathrm{Duration}\;(\mathrm{ms})}{\mathrm{Bout}\;\mathrm{Length}\;(\mathrm{licks})}.$$

The LIQ HD systems used in this study were upgraded to log data in an identical structure to LIQ PARTI.

### LIQ HD operation (single housed)

The single-housed LIQ HD system was built and operated as previously described (Petersen et al., 2023), with the exception that the capacitive sensor settings were optimized for more accurate lick detection to closely match the lick detection settings of LIQ PARTI. A detailed LIQ HD step-by-step guide, along with the Arduino code and 3D models, can be found at https://github.com/nickpetersen93/LIQ_HD.

### LIQ PARTI video recording validation

In a separate cohort of 10 male and 10 female mice across four cages, 10 min video recordings were taken of each cage to validate RFID tracking accuracy. The tail of each mouse was marked with a permanent marker of a different color in order to manually score the videos. Two to three mice per cage were randomly selected to receive a 200 µl intraperitoneal injection of hypertonic saline (2 M NaCl) to induce thirst 15 min before video recording (Augustine et al., 2018; Pool et al., 2020). The remaining mice in each cage received an intraperitoneal injection of 200 µl of normal saline (0.9% NaCl). During each video recording, both LIQ PARTI bottles contained water, and the Arduino activity was simultaneously recorded using the Arduino IDE serial monitor. Videos were manually scored alongside the Arduino activity to determine the accuracy of RFID tag identification during each recorded drinking bout. A representative video was manually annotated using Premiere Rush (Adobe). The percentage of bouts that were correctly identified was calculated by dividing the total number of recorded drinking bouts by the number of drinking bouts where the video-captured mouse (identified by tail color) matched the correct RFID number.

### Two-bottle voluntary ethanol choice task

To assess volitional ethanol intake in group-housed mice, a continuous access two-bottle choice task was used (Hodge et al., 1999; Centanni et al., 2019; Winters et al., 2021). Specifically, 10 male and 10 female mice were group housed with LIQ PARTI devices across four cages with 5 mice each. Following habituation to the animal facility and RFID tag implant surgery, mice were given a 7 d LIQ PARTI habituation period, during which both bottles contained only water. No measurements were taken during this time. Mice then received 7 d of water only in both bottles, followed by 3 d of 3% ethanol and water, 7 d of 7% ethanol and water, and 4 weeks of 10% ethanol and water (Hodge et al., 1999; Centanni et al., 2019; Winters et al., 2021). The mice were then singly housed with the LIQ HD system for 3 weeks with 10% ethanol and water. During social isolation recordings, the 20 mice were evenly divided between two separate identical LIQ HD systems to accommodate 20 cages. Lastly, the mice were returned to grouped housing with their previous cage mates with the LIQ PARTI system for two 8 d recording periods with 10% ethanol and water. For both LIQ PARTI and LIQ HD systems, experimenters manually changed cages, weighed bottles, and weighed mice at the end of each 3–8 d recording period. Cages were changed on Fridays (start of water week and 3% ethanol) and on Mondays (start of 7% ethanol and 10% ethanol weeks). The last cage change occurred on a Tuesday due to the 8-d-long recording period. Bottle placements were also swapped each time bottles were weighed to account for potential side biases. Recordings were resumed immediately after weight measurements were obtained and cages were changed.

### Statistical analysis

Data was first extracted and processed with a custom MATLAB script. The data were subdivided into critical time periods for statistical analysis. Unless otherwise stated, data were binned into 1 h bins. The estimated volume consumed was calculated by multiplying the lick number by 1.4 µl as determined by the validation correlations (Extended Data Fig. 1-2*F*). The estimated ethanol intake was calculated from the estimated volume consumed and the average weight of each mouse during the experiment. For the calculation of bottle preference over time, the binned data were smoothed with a moving average with a sliding window length of 12. Statistical analyses were performed with Prism 10 (GraphPad) and RStudio. For the individual bout data (Extended Data Figs. 1-4, 2-1), definitive outliers were removed using the ROUT method (*Q* = 0.1%). Pearson’s correlation coefficients and simple linear regressions were computed for all correlation pairs. *t* tests and ANOVAs with corrections for multiple comparisons were performed as indicated in the figure legends. Significance was defined as *p < 0.05, **p < 0.01, ***p < 0.001, and ****p < 0.0001.

### Code accessibility

The code/software described in the paper is freely available online at https://github.com/nickpetersen93/LIQ_PARTI. The code is available as Extended Data Fig. 1-1.

10.1523/ENEURO.0234-24.2024.d1 Data 1-1 LIQ PARTI Arduino source code used in this study. Download Data 1-1, ZIP file.

10.1523/ENEURO.0234-24.2024.d2 Data 1-2 Manually annotated representative video recording (shown here at 2x speed) for LIQ PARTI RFID-tracking validation. Mice, same-sex and five per cage, were implanted with a glass capsule RFID tag subcutaneously in the scruff of the neck and their tails were marked with different colors for video tracking. In this recording, three of the mice were injected with hypertonic saline, to induce thirst, and two mice were injected with normal saline 15 minutes before starting the recording. The mice were then placed in a clean cage with the LIQ PARTI system containing water in both bottles and allowed to explore the cage and consume water from either bottle for 10 minutes. The annotated circle icons indicate when an RFID tag was scanned, the water drop icons indicate when a drinking bout was identified, and the floppy disk save icon indicates when a drinking bout was saved to the SD card. The icon color matches the color of the mouse’s tail that corresponds to that mouse’s RFID number. The Arduino IDE serial monitor is displayed at the bottom of the video and displays real-time information. Download Data 1-2, MP4 file.

## Results

### LIQ PARTI accurately detects drinking bouts in a group-housed environment

We first validated the accuracy of LIQ PARTI to correctly identify RFID-tagged mice and to accurately record drinking behavior. A pilot cohort of 10 male and 10 female C57Bl/6J mice across four cages (5 same-sex mice each) were implanted with RFID tags under the scruff and allowed to recover and habituate to the LIQ PARTI system for at least 1 week. These mice were then used for video-scoring validation where a random subset of 12 mice (3 per cage) were injected with hypertonic saline to induce thirst (Augustine et al., 2018; Pool et al., 2020). The remaining mice were injected with normal saline as a control. Mice were placed in their home cage with the LIQ PARTI device, with both bottles containing water, 15 min after hypertonic saline injections, and were video recorded alongside the Arduino IDE serial monitor to time-lock RFID scans and drinking bouts for 10 min. As expected, mice injected with hypertonic saline had significantly more drinking events than mice injected with normal saline, which had no drinking bouts during the recording sessions (Extended Data Fig. 1-2*A–D*). There were 82 drinking bouts recorded throughout the 10 min videos across the four cages. LIQ PARTI attributed drinking events to the correct mouse for 81/82 of drinking bouts, representing a 99% accuracy rate (Extended Data Fig. 1-2*E*). See Extended Data Figure 1-2 for a fully annotated representative video recording.

**Figure 1.**
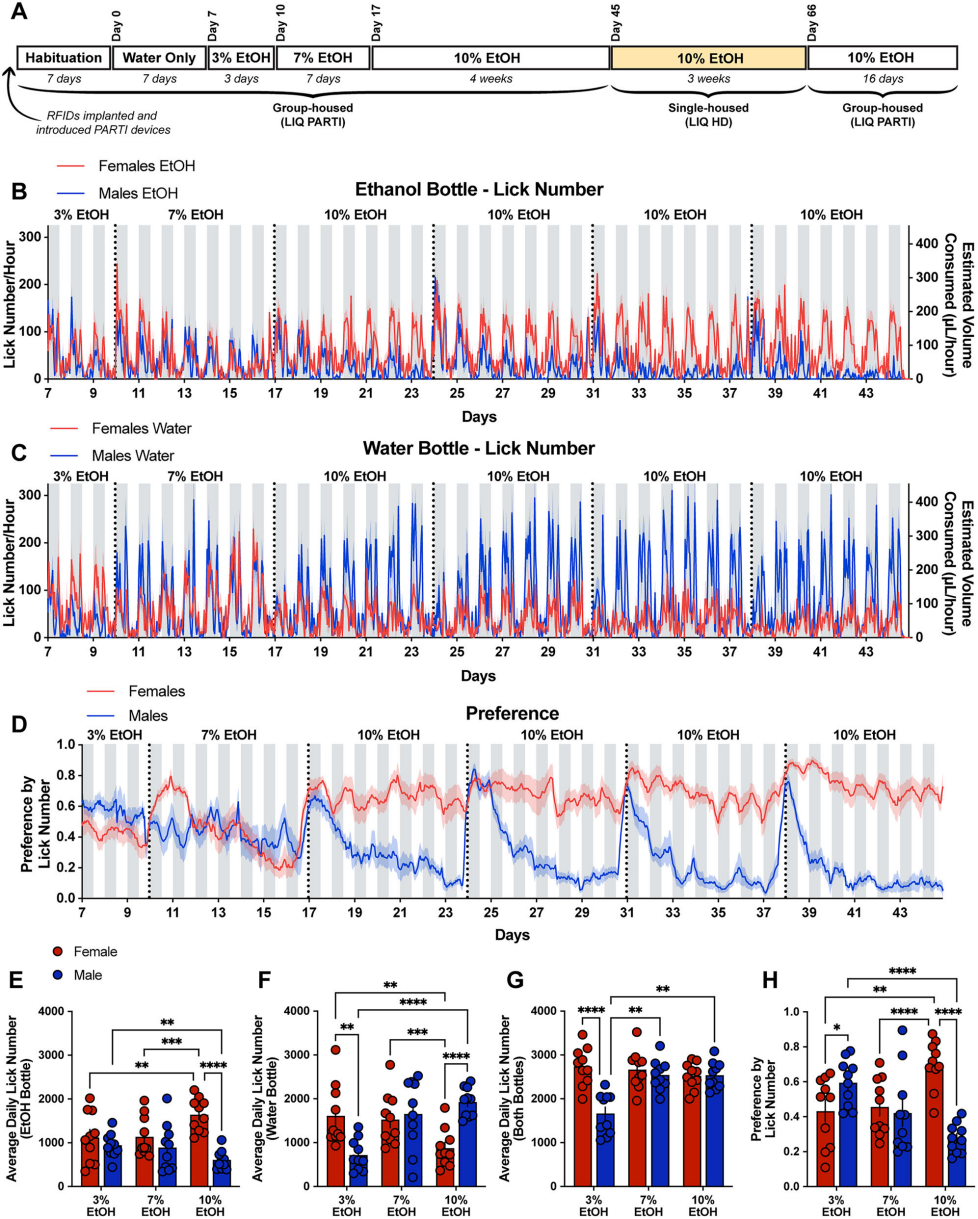
Female mice form a significantly higher preference for ethanol compared with males in a group-housed continuous access two-bottle choice task, and males display an undulating pattern of ethanol preference that aligns with cage-changing days. ***A***, Timeline of experimental setup. Ten male and 10 female mice across four cages were implanted with RFID tags under the scruff of the neck and given 7 d to recover and habituate with the LIQ PARTI devices. Mice were then exposed to a continuous two-bottle choice paradigm with water only for 1 week, 3% ethanol and water for 3 d, 7% ethanol and water for 1 week, and 10% ethanol and water for 4 weeks. Mice were then socially isolated with the LIQ HD system for 3 weeks and then returned to group housing with LIQ PARTI for 16 d. Lick number per hour and estimated volume consumed from male and female mice at the ethanol bottle (***B***) and water bottle (***C***) throughout ethanol exposure with LIQ PARTI prior to social isolation. ***D***, Ethanol preference score calculated by lick number in male and female mice throughout ethanol exposure with LIQ PARTI prior to social isolation. ***E***, Male and female mice had a similar number of average daily licks at the ethanol bottle during exposure to 3 and 7% ethanol, and female mice compared with males had a significantly higher number of licks at the ethanol bottle during exposure to 10% ethanol (repeated-measures two-way ANOVA, with the Geisser–Greenhouse correction and Tukey's multiple-comparisons test; significant main effect of sex *p* < 0.01 and significant interaction effect of sex × time *p* < 0.0001). ***F***, Female mice compared with males had significantly more licks at the water bottle during exposure to 3% ethanol, and male mice had significantly more licks at the water bottle during exposure to 10% ethanol compared with females (repeated measures two-way ANOVA, with the Geisser–Greenhouse correction and Tukey's multiple-comparisons test; significant main effect of time *p* < 0.05 and significant interaction effect of sex × time *p* < 0.0001). ***G***, Male and female mice show similar levels of average total lick number per day (both bottles) throughout ethanol exposure, except for during 3% ethanol and water where males displayed significantly fewer licks than females (repeated-measures two-way ANOVA, with the Geisser–Greenhouse correction and Tukey's multiple-comparisons test; significant main effect of sex *p* < 0.01 and time *p* < 0.01, significant interaction effect of sex × time *p* < 0.0001). ***H***, Male mice have a significantly higher preference for ethanol during exposure to 3% ethanol and water, but they significantly reduce their preference through exposure to 10% ethanol (repeated-measures two-way ANOVA, with the Geisser–Greenhouse correction and Tukey's multiple-comparisons test; significant interaction effect of sex × time *p* < 0.0001). Female mice have a significantly higher preference for ethanol than males during exposure to 10% ethanol with LIQ PARTI prior to social isolation. The solid lines (***B–D***) represent the mean lick number and estimated volume consumed in 1 h bins, and the red/blue shaded areas represent ±SEM. Gray-shaded areas represent the dark cycle and vertical dotted lines represent when cages were cleaned, when bottles were weighed, and when bottle positions were swapped. Error bars (***E–H***) represent ±SEM (*n* = 10 mice per group and reported as individual mice). *p < 0.05, **p < 0.01, ****p < 0.0001. See Extended Data Figure 1-1 for the LIQ PARTI wiring diagram and schematic, Extended Data Figure 1-1 for the LIQ PARTI Arduino source code, Extended Data Figure 1-2 for a representative annotated validation video, Extended Data Figure 1-2 for the LIQ PARTI video validation and correlation data, Extended Data Figure 1-3 for the water week lick data, Extended Data Figure 1-4 for the water week individual bout data, and Extended Data Figure 1-5 for the estimated hourly and daily ethanol intake (g/kg).

To determine the accuracy of LIQ PARTI to record drinking behavior during continuous access ethanol exposure, we correlated whole-cage lickometry data with experimenter-measured bottle weights. Specifically, we correlated the total lick number, lick duration, bout number, and bout duration detected from each bottle from each recording session to the independently measured volume consumed. Ten male and 10 female mice across four same-sex cages were implanted with RFID tags and allowed to habituate to the LIQ PARTI devices for 1 week. The mice were then given 1 week of water only, 3 d of 3% ethanol and water, 1 week of 7% ethanol and water, and 4 weeks of 10% ethanol and water (Hodge et al., 1999; Centanni et al., 2019; Winters et al., 2021). After this group-housed phase, the mice were then transferred to individual cages with the LIQ HD system for socially isolated drinking for 3 weeks with access to 10% ethanol and water before being returned to the group-housed LIQ PARTI system for 2 weeks, again with access to 10% ethanol and water (Fig. 1*A*). The measured volume change by weight during all group-housed recording sessions, irrespective of fluid type or ethanol exposure, was correlated to the various LIQ parameters to validate the accuracy of whole-cage intake behavior (Extended Data Fig. 1-2*F–M*). We observed a strong, significant correlation between total lick number and volume change (*R*^2^ = 0.9357; *F*_(1,54) _= 785.5; *p* < 0.0001; Extended Data Fig. 1-2*F*), total lick duration and volume change (*R*^2^ = 0.8724; *F*_(1,54) _= 369.1; *p* < 0.0001; Extended Data Fig. 1-2*G*), total bout number and volume change (*R*^2^ = 0.7742; *F*_(1,54) _= 185.2; *p* < 0.0001; Extended Data Fig. 1-2*H*), and total bout duration and volume change (*R*^2^ = 0.9446; *F*_(1,54) _= 921.5; *p* < 0.0001; Extended Data Fig. 1-2*I*). We observed a strong, significant correlation between the preference score calculated by the total lick number and the preference by volume change (*R*^2^ = 0.9809; *F*_(1,26) _= 1,339; *p* < 0.0001; Extended Data Fig. 1-2*J*), preference by total lick duration and preference by volume change (*R*^2^ = 0.9668; *F*_(1,26) _= 756.1; *p* < 0.0001; Extended Data Fig. 1-2*K*), preference by total bout number and preference by volume change (*R*^2^ = 0.9713; *F*_(1,26) _= 881.2; *p* < 0.0001; Extended Data Fig. 1-2*L*), and preference by total bout duration and preference by volume change (*R*^2^ = 0.9860; *F*_(1,26) _= 1,837; *p* < 0.0001; Extended Data Fig. 1-2*M*). The fitted regression model for the correlation between lick number and volume change is *Y* = 719.5*X −3,153 (slope 95% CI, 668.0–770.9). Thus, on average, the mice took 720 licks to drink 1 ml of fluid or 1.4 μl per lick. This is consistent with our previously published LIQ HD system (Petersen et al., 2023). Lick number will be used throughout this paper as a proxy for fluid consumption. Altogether, LIQ PARTI identifies RFID-tagged mice and drinking behavior with high fidelity.

10.1523/ENEURO.0234-24.2024.f1-1 Figure 1-1 LIQ PARTI wiring diagram and build. A, LIQ PARTI electronic parts and wiring diagram including an Arduino Mega, two RFID readers, an MPR121 capacitive touch board, a touchscreen shield, a data logger shield, and conductive copper foil tape. B, 3D rendering of LIQ PARTI disassembled components, including 3D-printed parts, rubber stoppers with sippers, RFID readers, and Arduino microcontroller. C, Glass capsule RFID tag with dimensions and representative illustration of the RFID tag to scale (estimated) with a mouse. More detailed information can be found at (https://github.com/nickpetersen93/LIQ_PARTI). Download Figure 1-1, TIF file.

**Figure 2.**
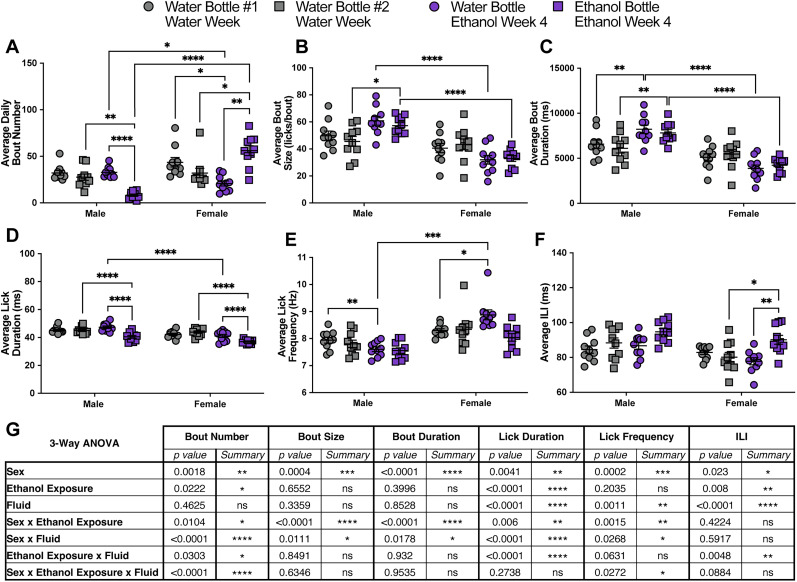
Exposure to ethanol significantly alters drinking microstructure in a sex- and bottle-specific manner in group-housed mice. Average daily bout number (***A***), bout size (***B***), bout duration (***C***), lick duration (***D***), lick frequency (***E***), and ILI (***F***) at the water and ethanol bottles for males and females during the water week (both bottles with water) and during the fourth week of 10% ethanol exposure (repeated-measures three-way ANOVA, with the Geisser–Greenhouse correction and Šidák multiple-comparisons test). ***G***, Three-way ANOVA results for the microstructure parameters showing the main effects of sex, ethanol exposure, fluid, and interactions. Error bars represent ±SEM (*n* = 10 mice per group and reported as individual mice). *p < 0.05, **p < 0.01, ***p < 0.001, ****p < 0.0001. See Extended Data Figure 2-1 for the individual bout data during the fourth week of 10% ethanol exposure.

10.1523/ENEURO.0234-24.2024.f1-2 Figure 1-2 LIQ PARTI accurately identifies individual mice in a group-housed environment and lick and bout detection closely correlates with bottle weight measurements. Total lick number (A), lick duration (B), bout number (C), and bout duration (D) of mice injected with hypertonic saline (HS) or normal saline (NS) during the LIQ PARTI video recording of water drinking for the validation experiment (unpaired t-test; n = 12 mice HS group; n = 8 mice NS group). E, The percentage of video-recorded drinking bouts that were attributed by LIQ PARTI to the correct RFID-tagged mouse. Correlation between whole-cage total lick number and volume change (F), total lick duration and volume change (G), total bout number and volume change (H), and total bout duration and volume change (I) for each recording period. Correlation between preference calculated by total lick number and preference calculated by volume change (J), preference by total lick duration and preference by volume change (K), preference by total bout number and preference by volume change (L), and preference by total bout duration and preference by volume change (M) for each recording period. (F-M) includes compiled data from all LIQ PARTI bottle measurements, irrespective of fluid type or ethanol exposure, from the 3-8 day recording periods. In correlation graphs, solid lines represent a fitted simple linear regression model, and dashed lines denote 95% confidence intervals. Data in A-E are reported as individual mouse values and F-M are reported as whole-cage values. ****p < 0.0001. Download Figure 1-2, TIF file.

**Figure 3.**
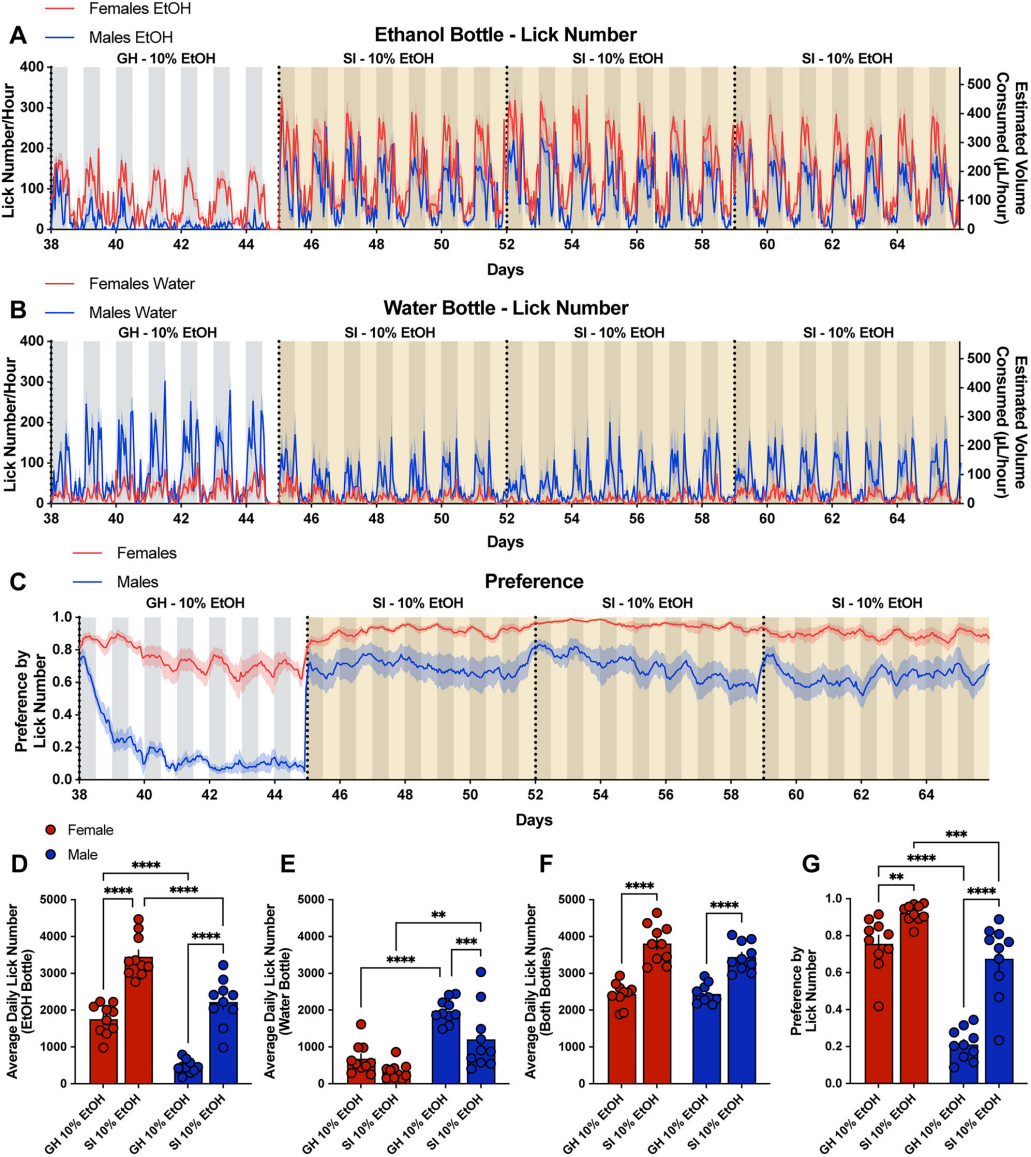
Social isolation in the LIQ HD system significantly increases ethanol preference and total consumption in male and female mice and changes the overall ethanol preference pattern over time in males. Lick number per hour and estimated volume consumed from male and female mice at the ethanol bottle (***A***) and water bottle (***B***) throughout the last week of 10% ethanol exposure with the group-housed (GH) LIQ PARTI system and through social isolation (SI) with LIQ HD with 10% ethanol and water. ***C***, Ethanol preference score calculated by lick number in male and female mice throughout the last week of 10% ethanol exposure with the group-housed LIQ PARTI system and through social isolation with LIQ HD with 10% ethanol and water. ***D***, Both male and female mice significantly increased their average daily lick number at the ethanol bottle during social isolation compared with grouped housing, and female mice had significantly higher licks at the ethanol bottle compared with males during social isolation and grouped housing (repeated-measures two-way ANOVA, Šidák multiple-comparisons test; significant main effect of sex *p* < 0.0001 and housing *p* < 0.0001). ***E***, Males, but not females, significantly decreased their average daily number at the water bottle during social isolation compared with grouped housing, and males had significantly higher licks at the water bottle compared with females during social isolation and grouped housing (repeated-measures two-way ANOVA, Šidák multiple-comparisons test; significant main effect of sex *p* < 0.0001 and housing *p* < 0.0001). ***F***, Both male and female mice display an increase in the average total lick number per day (both bottles) during social isolation with no significant difference between male and female mice (repeated-measures two-way ANOVA, Šidák multiple-comparisons test; significant main effect of housing *p* < 0.0001, significant interaction effect of sex × housing *p* < 0.05). ***G***, Both male and female mice have an increased preference for ethanol during social isolation and female mice have a higher ethanol preference than males during group housing with LIQ PARTI and social isolation with LIQ HD (repeated-measures two-way ANOVA, Šidák multiple-comparisons test; significant main effect of sex *p* < 0.0001 and housing *p* < 0.0001, significant interaction effect of sex × housing *p* < 0.0001). The solid lines (***A–C***) represent the mean lick number in 1 h bins, and the red/blue shaded areas represent ±SEM. Gray-shaded areas represent the dark cycle and vertical dotted lines represent when cages were cleaned, when bottles were weighed, and when bottle positions were swapped. Yellow-shaded regions indicate periods of social isolation with LIQ HD. Error bars (***D–G***) represent ±SEM (*n* = 10 mice per group and reported as individual mice). **p < 0.01, ***p < 0.001, ****p < 0.0001.

10.1523/ENEURO.0234-24.2024.f1-3 Figure 1-3 Group-housed male and female mice consume water at similar rates when given continuous access to two water bottles, and female mice show a significantly higher average lick frequency and lick duration compared to males at baseline. Lick number per hour and estimated volume consumed from male and female mice at the left water bottle (A) and the right water bottle (B) throughout the water-only week with the group-housed LIQ PARTI system. C, Bottle preference score calculated by lick number at each water bottle in male and female mice throughout the water-only week. The average lick number per day (D), average preference by lick number (E), average daily bout number (F), average bout size (G), average bout duration (H), average lick duration (I), average lick frequency (J), and average ILI (K) were calculated based on total values at both bottles and compared between males and females during the water-only week with LIQ PARTI (unpaired t-test). The solid lines (A-C) represent the mean lick number in 1-h bins, and the red/blue shaded areas represent ±SEM. Gray-shaded areas represent the dark cycle. Error bars (D-K) represent ±SEM. (n = 10 mice per group and reported as individual mice). *p < 0.05. Download Figure 1-3, TIF file.

**Figure 4.**
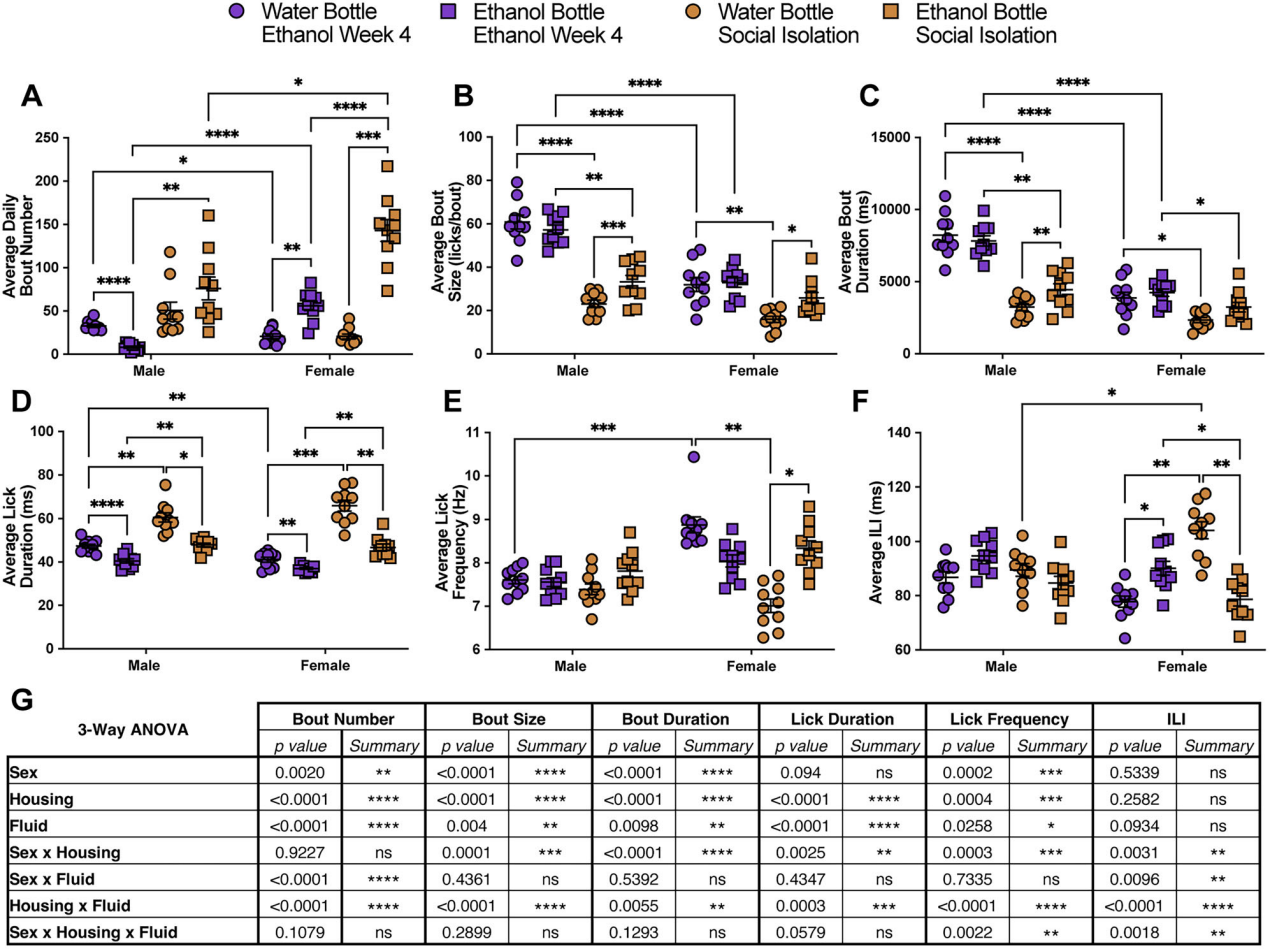
Social isolation significantly alters drinking microstructure in a sex- and bottle-specific manner. Average daily bout number (***A***), bout size (***B***), bout duration (***C***), lick duration (***D***), lick frequency (***E***), and ILI (***F***) at the water and ethanol bottles for males and females during the fourth week of group-housed 10% ethanol exposure and during social isolation with 10% ethanol (averaged across the 3 weeks; repeated-measures three-way ANOVA, with the Geisser–Greenhouse correction and Šidák multiple-comparisons test). ***G***, Three-way ANOVA results for the microstructure parameters showing the main effects of sex, housing, fluid, and interactions. Error bars represent ±SEM (*n* = 10 mice per group and reported as individual mice). *p < 0.05, **p < 0.01, ***p < 0.001, ****p < 0.0001.

10.1523/ENEURO.0234-24.2024.f1-4 Figure 1-4 Bout microstructure is significantly different across sex, cage, and individual mice during the water-only week. Individual bout duration (A), individual bout size (B), bout lick frequency (C), and individual lick duration (D) for each bout during the water-only week (both water bottles combined). All bout microstructure parameters show significant differences between individual mice in the same cage (one-way ANOVA with Tukey’s test for multiple comparisons for within-cage comparisons). E, All bout microstructure parameters differ significantly across cages and between male and female mice (two-way nested ANOVA, main factor of sex, cage nested into sex). Each data point represents an individual bout that occurred during the water-only recording period. The number of bouts ranges from n = 302 to n = 934. The vertical dotted line separates mice in each cage. The solid horizontal line represents the sample means. Download Figure 1-4, TIF file.

**Figure 5.**
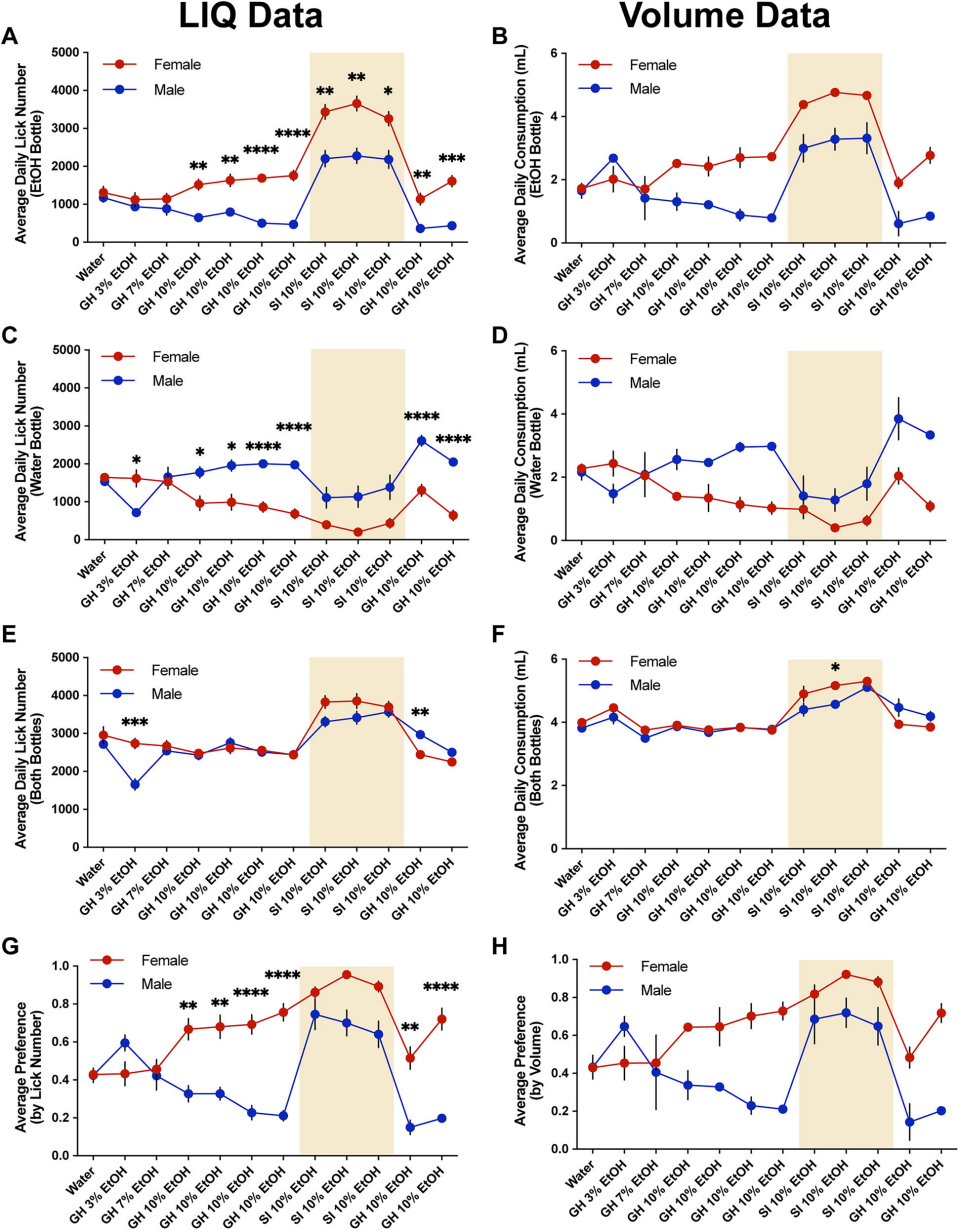
Summary of LIQ and volume measurement data throughout the two-bottle choice drinking paradigm. ***A***, Average daily lick number at the ethanol bottle with a significant main effect of time (*p* < 0.0001) and sex (*p* < 0.0001) and a significant interaction effect (time × sex, *p* < 0.0001). ***B***, Average daily consumption at the ethanol bottle with a significant main effect of time (*p* < 0.01) and sex (*p* < 0.05) and a significant interaction effect (time × sex, *p* < 0.01). ***C***, Average daily lick number at the water bottle with a significant main effect of time (*p* < 0.0001) and sex (*p* < 0.001) and a significant interaction effect (time × sex, *p* < 0.0001). ***D***, Average daily consumption at the water bottle with a significant main effect of sex (*p* < 0.05) and a significant interaction effect (time × sex, *p* < 0.01). ***E***, Average daily lick number at both bottles combined with a significant main effect of time (*p* < 0.0001) and a significant interaction effect (time × sex, *p* < 0.0001). ***F***, Average daily consumption at both bottles combined with a significant main effect of time (*p* < 0.01) and sex (*p* < 0.01). ***G***, Average preference by lick number with a significant main effect of time (*p* < 0.0001) and sex (*p* < 0.0001) and a significant interaction effect (time × sex, *p* < 0.0001). ***H***, Average preference by average volume consumed with a significant main effect of time (*p* < 0.05) and sex (*p* < 0.05) and a significant interaction effect (time × sex, *p* < 0.01). Volume measurement data was calculated as the average across all animals per cage (LIQ data: repeated-measures mixed-effects model, with the Geisser–Greenhouse correction and Šidák multiple-comparisons test for female versus male at each time point. Volume data: repeated-measures two-way ANOVA, with the Geisser–Greenhouse correction and Šidák multiple-comparisons test for female vs male at each time point). Yellow-shaded regions indicate periods of social isolation with LIQ HD. Error bars represent ±SEM (LIQ data *n* = 10 mice per group, except males post-SI week 2 *n* = 5, and are reported as individual mouse values; volume data *n* = 2 cages per group and are reported as whole-cage values). *p < 0.05, **p < 0.01, ***p < 0.001, ****p < 0.0001.

10.1523/ENEURO.0234-24.2024.f1-5 Figure 1-5 Estimated ethanol intake throughout the experiment. A, Estimated hourly ethanol intake over time across all ethanol-exposed recording periods. B, Estimated daily ethanol intake over time across all recording periods (repeated measures two-way ANOVA with the Geisser–Greenhouse correction and Šidák multiple comparisons test for female versus male at each time point; significant main effect of Time p<0.0001 and Sex p<0.0001, and significant interaction effect of Time x Sex p<0.0001; n = 10 mice per group). Yellow-shaded regions indicate periods of social isolation with LIQ HD. Error bars represent ±SEM. ***p < 0.001, ****p < 0.0001. Download Figure 1-5, TIF file.

### Male and female mice display significant differences in ethanol preference, drinking patterns, and microstructure in a group-housed *ad libitum* home cage drinking environment

In the same cohort, we next utilized LIQ PARTI to evaluate the ethanol drinking behavior of individual C57Bl/6J mice during the continuous access voluntary two-bottle choice paradigm (Hodge et al., 1999; Centanni et al., 2019; Winters et al., 2021) in a group-housed environment. As described above, the mice were given access to water in both bottles for 1 week (Extended Data Fig. 1-3). We did not observe any significant differences in the total number of licks (Extended Data Fig. 1-3*A,B,D*) or bottle preference (Extended Data Fig. 1-3*C,E*) between the male and female group-housed mice, suggesting male and female mice consume water at similar rates when given continuous access to two water bottles. On average, female mice displayed a significantly higher average lick duration (Extended Data Fig. 1-3*I*) and average lick frequency (licks per second; Extended Data Fig. 1-3*J*) than males, with no significant differences in average daily bout number, bout size (licks per bout), bout duration, or ILI (Extended Data Fig. 1-3*F–H,K*). However, analyses considering all drinking bouts during the water week revealed a significant difference between sex and cage for individual bout duration, bout size, lick frequency, and lick duration (Extended Data Fig. 1-4*A–E*). We additionally observed significant individual differences between mice in each cage across all bout microstructure parameters (Extended Data Fig. 1-4*A–D*).

The group-housed mice were then given access to 3% ethanol and water for 3 d, 7% ethanol and water for 1 week, and 10% ethanol and water for 4 weeks (Fig. 1). Male and female mice had a similar number of average daily licks at the ethanol bottle during exposure to 3 and 7% ethanol, and female mice had a significantly higher number of licks at the ethanol bottle compared with males during exposure to 10% ethanol (Fig. 1*B*,*E*). Female mice had significantly more licks at the water bottle compared with males during exposure to 3% ethanol, and male mice had significantly more licks at the water bottle during exposure to 10% ethanol compared with females (Fig. 1*C*,*F*). Male and female mice had a similar number of average total licks per day (both bottles), except during access to 3% ethanol where male mice had significantly fewer licks (Fig. 1*B*,*C*,*G*). However, female mice developed a significantly higher preference for ethanol during access to 10% ethanol compared with 3 and 7% ethanol and compared with males at 10% ethanol (Fig. 1*D*,*H*). Throughout the experiment, except during exposure to 3% ethanol, female mice had a significantly higher estimated ethanol intake (g/kg) compared with male mice (Extended Data Fig. 1-5*A,B*). Interestingly, males showed a gradual decrease in the average preference for ethanol throughout ethanol exposure, with significantly higher ethanol preference during 3% ethanol compared with females and compared with themselves at 10% ethanol. Dirty cages were exchanged each week throughout the experiment, which corresponded to within-week fluctuations in the preference for ethanol in male mice only. With the advantage of high temporal resolution with LIQ PARTI, we observed that male mice displayed an undulating pattern of ethanol preference with a significantly higher preference for ethanol, at similar levels to the females, at the beginning of each recording week period (Fig. 1*D*). These spikes in male ethanol preference occur in tandem with bedding changes and diminish over 24–48 h.

In addition to differences in ethanol preference between male and female group-housed mice, the animals showed significant differences in bout microstructure that were ethanol exposure, sex, and fluid (ethanol or water) dependent (Fig. 2). Data during the water-only week were compared with data from the fourth week of 10% ethanol exposure. Compared with water-only access, during exposure to 10% ethanol, male mice significantly decreased their average daily bout number at the ethanol bottle while female mice increased their average daily bout number at the ethanol bottle and decreased at the water bottle (Fig. 2*A*). Compared with the male mice during 10% ethanol exposure, female mice had a significantly lower average daily bout number at the water bottle and a significantly higher bout number at the ethanol bottle (Fig. 2*A*). Male mice significantly increased their bout size at the ethanol bottle and bout duration at the water and ethanol bottle compared with the water-only week (Fig. 2*B*,*C*). Female mice did not significantly change their bout size or duration at either bottle in response to ethanol exposure and had significantly smaller and shorter bouts at both bottles compared with males during 10% ethanol (Fig. 2*B*,*C*). Both male and female mice displayed a decrease in the average lick duration at the ethanol bottle compared with the water bottle during 10% ethanol exposure and compared with the water week (Fig. 2*D*). Female mice also had a decreased average lick duration at the water bottle compared with male mice during exposure to 10% ethanol (Fig. 2*D*). Male mice significantly decreased their lick frequency at the water bottle during exposure to 10% ethanol and had a significantly slow lick frequency during ethanol exposure at the water bottle compared with females (Fig. 2*E*). In contrast, females significantly increased their lick frequency at the water bottle during exposure to 10% ethanol (Fig. 2*E*). The ILI of male mice did not significantly change at either bottle in response to ethanol exposure, but female mice showed a significant increase in the ILI at the ethanol bottle compared with the water week and compared with the water bottle during 10% ethanol exposure (Fig. 2*F*). Utilizing analyses considering all drinking bouts during the fourth week of 10% ethanol (both bottles combined), we observed a significant difference between sex and cage for individual bout duration, bout size, lick frequency, and lick duration (Extended Data Fig. 2-1*A–E*). Additionally, there were significant individual differences between mice in each cage across all bout microstructure parameters (Extended Data Fig. 2-1*A–D*). Altogether, these data demonstrate significant changes to bout microstructure following exposure to chronic, continuous access ethanol in group-housed mice that are dependent on both sex and the fluid that is being consumed (Fig. 2*G*).

10.1523/ENEURO.0234-24.2024.f2-1 Figure 2-1 Bout microstructure is significantly different across sex, cage, and individual mice during the fourth week of 10% ethanol exposure. Individual bout duration (A), individual bout size (B), bout lick frequency (C), and individual lick duration (D) for each bout during the fourth week of 10% ethanol (both water bottles combined). All bout microstructure parameters show significant differences between individual mice in the same cage (one-way ANOVA with Tukey’s test for multiple comparisons for within cage comparisons). E, All bout microstructure parameters differ significantly across cages and between male and female mice (two-way nested ANOVA, main factor of sex, cage nested into sex). Each data point represents an individual bout that occurred during the fourth week of 10% ethanol exposure. The number of bouts ranges from n = 231 to n = 810. The vertical dotted line separates mice in each cage. The solid horizontal line represents the sample means. Download Figure 2-1, TIF file.

### Social isolation enhances ethanol preference and alters drinking microstructure in a sex- and bottle-specific manner

To investigate the effect of social isolation on alcohol-drinking behavior and bout microstructure in male and female mice, the animals were transferred into individual cages equipped with the LIQ HD system (Petersen et al., 2023). The LIQ HD code was modified to enhance the lick detection of the capacitive sensor and to match the bout detection parameters to the LIQ PARTI system. To validate these changes, we correlated the total lick number at each bottle with the experimenter-measured volume change as well as the preference calculated by lick number with the preference by volume change. The data from the 3 weeks of social isolation were combined for these correlations. We observed a strong, significant correlation between total lick number and volume change (*R*^2^ = 0.9509; *F*_(1,117) _= 2,268; *p* < 0.0001; Extended Data Fig. 3-1*A*) and between preference by total lick number and preference by volume change (*R*^2^ = 0.8731; *F*_(1,58) _= 399.1; *p* < 0.0001; Extended Data Fig. 3-1*B*). The fitted regression model for the correlation between lick number and volume change is *Y* = 737.7*X −114.9 (slope 95% CI, 707.0–768.4), corresponding to an average of 738 licks per 1 ml of fluid or 1.4 μl per lick, which is consistent with our previous LIQ HD data (Petersen et al., 2023) and LIQ PARTI. The slopes of the fitted regression of total lick number versus volume change for LIQ HD and LIQ PARTI did not significantly differ (Extended Data Fig. 3-1*C*).

Ethanol drinking behavior substantially increased in both male and female mice when transferred to social isolation (Fig. 3). Compared with the fourth week of group-housed 10% ethanol, both male and female mice significantly increased their average total lick number per day across both the ethanol and the water bottle combined, and the average total lick number per day did not differ by sex (Fig. 3*A*,*B*,*F*). Both male and female mice significantly increased their average daily lick number at the ethanol bottle during social isolation compared with grouped housing, and female mice had significantly higher licks at the ethanol bottle compared with males during social isolation and grouped housing (Fig. 3*D*). Males, but not females, significantly decreased their average daily number at the water bottle during social isolation compared with grouped housing, and males had significantly higher licks at the water bottle compared with females during social isolation and grouped housing (Fig. 3*E*). Additionally, the preference for ethanol significantly increased during social isolation in both males and females but to a greater magnitude in males (Fig. 3*C*,*G*). However, this may be due to a ceiling effect in the females. Consistent with ethanol preference during group housing, females have a significantly greater preference for ethanol compared with males during social isolation (Fig. 3*C*,*G*).

10.1523/ENEURO.0234-24.2024.f3-1 Figure 3-1 LIQ HD and LIQ HD vs LIQ PARTI validation. Correlation between total lick number and volume change (A) and correlation between preference calculated total lick number and preference by volume change (B) for each recording period of social isolation with LIQ HD. C, comparison of correlations between total lick number and volume change for LIQ HD and LIQ PARTI. There is no significant difference between the fitted region slopes for the LIQ HD and LIQ PARTI systems. Solid lines represent a fitted simple linear regression model, and dashed lines denote 95% confidence intervals. LIQ PARTI data are reported as whole-cage values. Download Figure 3-1, TIF file.

Ethanol drinking bout microstructure was significantly altered in a housing-, sex-, and fluid-dependent manner. The average daily bout number at the ethanol bottle significantly increased for both male and female mice during social isolation compared with grouped housing, with female mice displaying significantly more average daily bouts at the ethanol bottle compared with males during grouped housing and social isolation (Fig. 4*A*). Prior to social isolation, male mice had significantly larger and longer bouts at both ethanol and water bottles compared with female mice (Fig. 4*B*,*C*). Following social isolation, male mice significantly decreased their bout size and bout durations at both the water and ethanol bottles to comparable levels to the female mice (Fig. 4*B*,*C*). Female mice significantly decreased their bout size and duration at the water bottle and decreased bout duration at the ethanol bottle. Both male and female mice had significantly larger bouts at the ethanol bottle compared with the water bottle during social isolation, and male mice had increased bout duration at the ethanol bottle (Fig. 4*B*,*C*). Male and female mice also showed an increased average lick duration at both the water and ethanol bottles compared with the group-housed setting (Fig. 4*D*). Additionally, both sexes had significantly longer lick duration at the water bottle compared with the ethanol bottle during both group housing and social isolation but to a greater magnitude during isolation (Fig. 4*D*). Average lick frequency and ILI did not significantly change in male mice in response to social isolation (Fig. 4*E*,*F*). However, female mice significantly reduced their lick frequency at the water bottle and had a significantly higher lick frequency at the ethanol bottle in comparison with the water bottle during social isolation (Fig. 4*F*). While female mice had a higher ILI at the ethanol bottle compared with the water bottle during group housing, the opposite effect was seen during social isolation. Female mice had an increased ILI at the water bottle and a decreased ILI at the ethanol bottle in response to social isolation (Fig. 4*F*). Taken together, social isolation leads to robust changes in bout microstructure in male and female ethanol-experienced mice that vary based on sex and fluid (Fig. 4*G*).

### Mice returned to a group-housed environment following social isolation displayed sex-specific changes in ethanol preference compared with pre-social isolation

**Figure 6.**
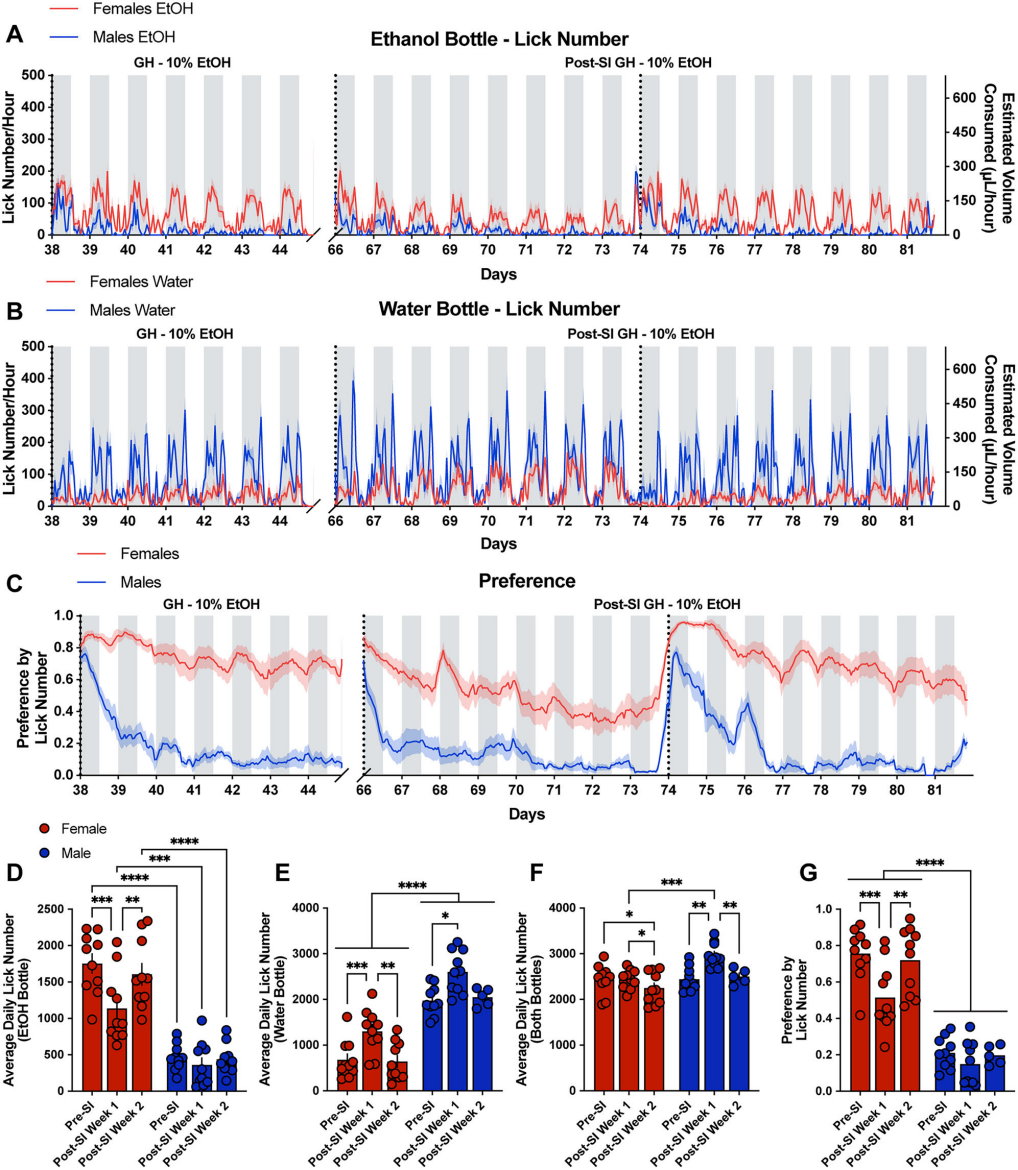
The average lick number per day modestly decreases in females and returns to baseline in males, and ethanol preference returns to baseline in both males and females during the return to a group-housed setting postisolation compared with the last previous group-housed week. Lick number per hour and estimated volume consumed from male and female mice at the ethanol bottle (***A***) and water bottle (***B***) throughout the last week of 10% ethanol exposure with the group-housed LIQ PARTI system and through postsocial isolation with 10% ethanol and water. ***C***, Ethanol preference score calculated by lick number in male and female mice throughout the last week of 10% ethanol exposure with the group-housed LIQ PARTI system and through postsocial isolation with 10% ethanol and water. Male mice resume the previously observed pattern of undulating ethanol preference that corresponds with cage-changing days. ***D***, Female mice, but not males, significantly decreased their average daily lick number at the ethanol bottle during the first week of postsocial isolation compared with presocial isolation but returned to presocial isolation levels by Week 2. Female mice had significantly more licks at the water bottle compared with males during all three time periods (repeated-measures mixed-effects model, Tukey's multiple-comparisons test; significant main effect of sex *p* < 0.0001 and housing *p* < 0.0001, significant interaction effect of sex × housing *p* < 0.01). ***E***, Both male and female mice significantly increased their daily average lick number at the water bottle during the first postsocial isolation week compared with presocial isolation but returned to presocial isolation levels by Week 2. Males have significantly more licks at the water bottle during all three time periods compared with females (repeated-measures mixed-effects model, Tukey's multiple-comparisons test; significant main effect of sex *p* < 0.0001 and housing *p* < 0.0001). ***F***, Female mice show no significant difference in the average total licks per day (both bottles) during the first postsocial isolation recording session, but modestly decrease during the second session. Male mice display a significantly greater average total lick number per day during with first recording session postsocial isolation with no significant difference during the second session compared with the last week of presocial isolation 10% ethanol (repeated-measures mixed-effects model, Tukey's multiple-comparisons test; significant main effect of sex *p* < 0.05 and housing *p* < 0.0001, significant interaction effect of sex × housing *p* < 0.001). ***G***, Female mice have a significantly lower ethanol preference during the first week of postsocial isolation compared with the last week of presocial isolation with 10% ethanol. Ethanol preference returns to presocial isolation levels in females during the second postsocial isolation recording session. Male mice immediately return to presocial isolation ethanol preference post-social (repeated-measures mixed-effects model, Tukey's multiple-comparisons test; significant main effect of sex *p* < 0.0001 and housing *p* < 0.001, significant interaction effect sex × housing *p* < 0.01). The solid lines (***A–C***) represent the mean lick number in 1 h bins, and the red/blue shaded areas represent ±SEM. Gray-shaded areas represent the dark cycle and vertical dotted lines represent when cages were cleaned, when bottles were weighed, and when bottle positions were swapped. Error bars (***D–G***) represent ±SEM (*n* = 10 mice per group, except males post-SI week 2 *n* = 5, and reported as individual mice). *p < 0.05, **p < 0.01, ***p < 0.001, ****p < 0.0001.

Following social isolation with the LIQ HD system, the mice were returned to group housing with their original cage mates and the LIQ PARTI systems to investigate whether social isolation-induced changes to drinking behavior persist after social reintegration. The mice were group-housed for two recording periods each 8 d long. A cage of male mice was excluded from the analysis of the second recording period due to user error when starting the system. However, bottle measurement data closely recapitulate the same conclusions (Fig. 5). Compared with the last group-housed 10% ethanol week presocial isolation, female mice, but not males, significantly decreased their average daily lick number at the ethanol bottle during the first week of postsocial isolation but returned to presocial isolation levels by Week 2, and female mice had significantly more licks at the water bottle compared with males during all three time periods (Figs. 5*A*,*B*, 6*A*,*D*). Both male and female mice significantly increased their daily average lick number at the water bottle during the first postsocial isolation week but returned to presocial isolation levels by Week 2, and male mice had significantly more licks at the water bottle during all three time periods compared with females (Figs. 5*C*,*D*, 6*B*,*E*). Female mice decreased their average total lick number per day (both bottles) back to presocial isolation levels and further decreased during the second recording period of group housing postsocial isolation compared with the last group-housed 10% ethanol week presocial isolation (Figs. 5*E*,*F*, 6*A*,*B*,*F*). Male mice continued to show an increase in the average total licks per day during the first period of postsocial isolation but returned to presocial isolation levels during the second recording period (Figs. 5*E*,*F*, 6*A*,*B*,*F*). Additionally, male mice showed a greater average total lick number per day compared with females during the first week of postsocial isolation group housing but returned to comparable levels by the second recording period (Figs. 5*E*,*F*, 6*A*,*B*,*F*). Ethanol preference was significantly lower overall in male mice versus female mice pre- and postsocial isolation (Figs. 5*A–D*,*G*,*H*, 6*C*,*G*), as previously reported (Fig. 1*D*,*H*). Male mice immediately reverted to presocial isolation ethanol preference and restarted the same undulating pattern of preference corresponding to cage changes (Figs. 5*A–D*,*G*,*H*, 6*C*,*G*). In contrast, female mice had a significantly lower ethanol preference postsocial isolation compared with presocial isolation but reverted to a similar level of preference by the second recording period (Figs. 5*A–D*,*G*,*H*, 6*C*,*G*).

**Figure 7.**
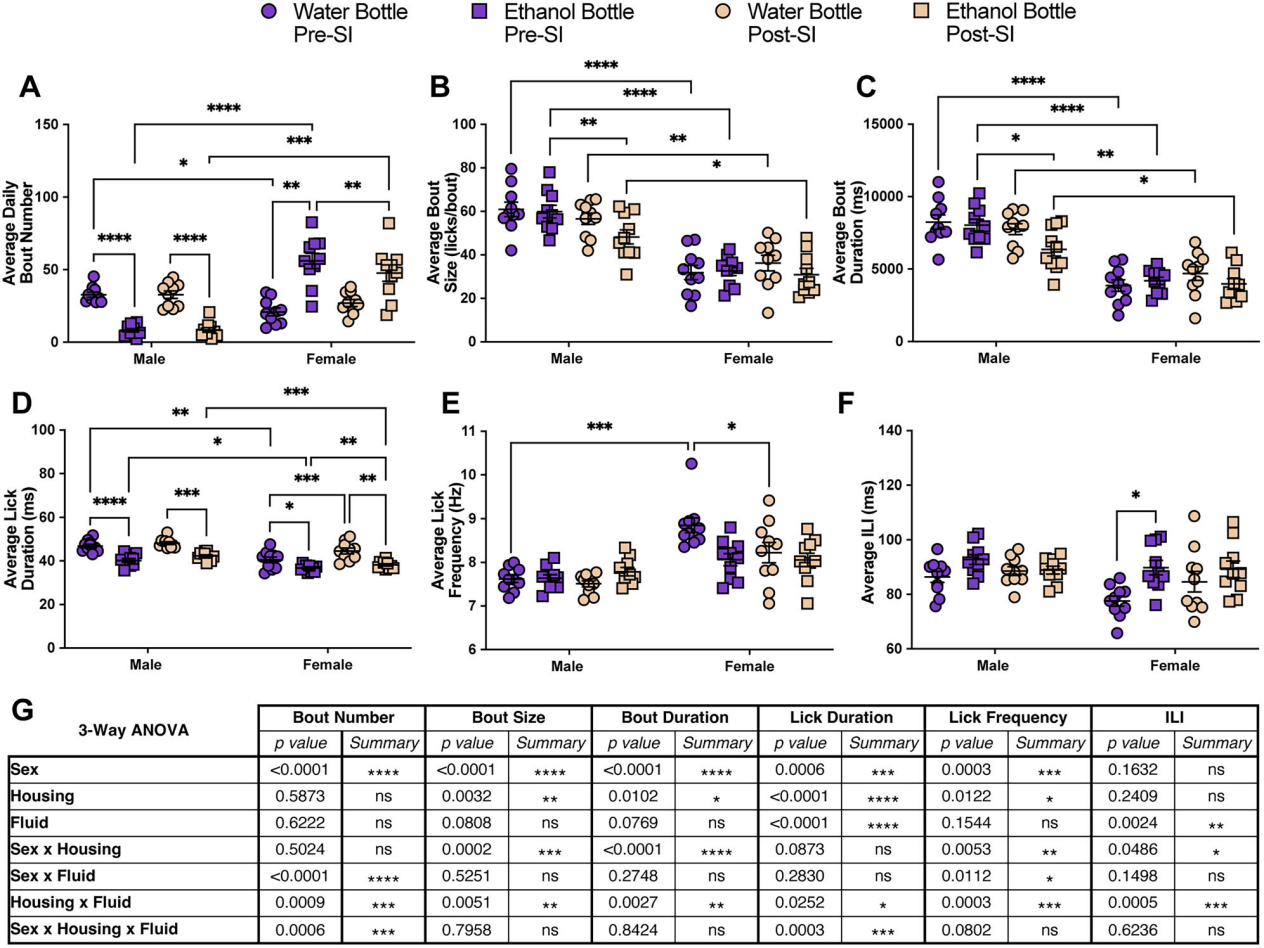
Social reintegration significantly alters drinking microstructure in a sex- and bottle-specific manner, which largely reverts to presocial isolation values. Average daily bout number (***A***), average bout size (***B***), bout duration (***C***), lick duration (***D***), lick frequency (***E***), and ILI (***F***) at the water and ethanol bottles for males and females during fourth week of group-housed 10% ethanol exposure (pre-SI) and during postsocial isolation with 10% ethanol (post-SI, averaged across the 2 recording periods; repeated-measures three-way ANOVA, with the Geisser–Greenhouse correction and Šidák multiple-comparisons test). ***G***, Three-way ANOVA results for the microstructure parameters showing the main effects of sex, housing, fluid, and interactions. Error bars represent ±SEM (*n* = 10 mice per group and reported as individual mice). *p < 0.05, **p < 0.01, ***p < 0.001, ****p < 0.0001. See Extended Data Figure 7-1 for the average daily microstructure over the course of the experiment.

Bout microstructure parameters mostly reverted to presocial isolation values with some exceptions (Fig. 7; Extended Data Fig. 7-1). While significant differences between sexes remained, male mice showed no significant difference in the average daily bout number at the water or ethanol bottles postsocial isolation compared with presocial isolation (Fig. 7*A*; Extended Data Fig. 7-1*A*). The average daily bout number at the water bottle for females also showed no significant difference after social isolation compared with presocial isolation, but female mice showed a moderate decrease in the average daily bout number at the ethanol bottle during postsocial isolation (Fig. 7*A*; Extended Data Fig. 7-1*A*). Male mice displayed a significant decrease in bout size and bout duration at the ethanol bottle compared with presocial isolation and female mice reverted to presocial isolation values (Fig. 7*B*,*C*; Extended Data Fig. 7-1*B,C*). In contrast, male mice reverted to presocial isolation average lick duration values at both bottles while female mice showed a small, but significant increase in the average lick duration at both the water and ethanol bottles postsocial isolation (Fig. 7*D*; Extended Data Fig. 7-1*D*). Lick frequency and ILI remained unchanged in the male mice (Fig. 7*E*,*F*; Extended Data Fig. 7-1*E,F*). Female mice had shown significant differences in lick frequency and ILI between the water and ethanol bottles during social isolation (Fig. 4*E*,*F*) but showed no significant differences between the bottles during postsocial isolation group housing with a significant decrease in lick frequency at the water bottle post-social isolation compared with pre-social isolation (Fig. 7*E*,*F*; Extended Data Fig. 7-1*E,F*). These data highlight that while social isolation induces substantial changes in alcohol drinking behavior in male and female mice, these behaviors are plastic and can essentially reverse following reintroduction to a social environment.

10.1523/ENEURO.0234-24.2024.f7-1 Figure 7-1 Exposure to ethanol and social isolation significantly alters drinking microstructure in a sex-, bottle, and housing-specific manner over time. Average daily bout number (A), bout size (B), bout duration (C), lick duration (D), lick frequency (E), and ILI (F) for male and female mice at the water and ethanol bottles. Lines represent the means in 24-h bins, and the red/blue shaded areas represent ±SEM. Yellow-shaded regions indicate periods of social isolation with LIQ HD and vertical dotted lines represent when cages were cleaned and bottle positions were swapped. (n = 10 mice per group, except males post-SI week 2 n = 5, and reported as individual mice). Download Figure 7-1, TIF file.

## Discussion

We utilized LIQ PARTI, a novel mouse home cage lickometer system to record two-bottle choice drinking behavior in a group-housed environment, to investigate sex differences and the effects of social environments on continuous access ethanol drinking and bout microstructure in C57Bl/6J mice. LIQ PARTI is built upon the same technology in our previously published LIQ HD system (Petersen et al., 2023) that uses capacitive-based touch sensors to detect single lick events. Each cage is controlled by a single Arduino MEGA microcontroller connected to a real-time clock plus data logging shield, a touchscreen shield, a MPR121 capacitive sensor breakout board, and two 125 kHz RFID readers. The two RFID readers lay inside the 3D-printed housing above two small tunnels that the mice must enter to reach each sipper, which allows the device to read an implanted RFID tag to determine which mouse is actively drinking from each sipper with 99% accuracy. The Arduino microcontroller records each drinking bout with a timestamp and automatically logs the number of licks, the bout duration, the total lick duration (contact time), the average lick frequency, and the average ILI of each bout to an SD card. We show that in addition to the high accuracy of RFID identification, LIQ PARTI records drinking activity and bottle preference with remarkably high accuracy, as measured through a correlation of LIQ activity and manually weighed bottle measurements (Extended Data Fig. 1-2). With this system, we observed that baseline measurements of water consumption between male and female group-housed mice are similar, except for a subtle, but significant increase in lick duration and frequency in female mice compared with males (Extended Data Fig. 1-3).

With the use of LIQ PARTI in a continuous access two-bottle choice ethanol paradigm, we show that male and female group-housed mice develop different preferences for ethanol and modulate their bout microstructure in contrasting ways. As previously reported throughout the literature, female C57Bl/6J mice develop a significantly higher preference for ethanol than males (Fig. 1; Middaugh et al., 1999; Lopez and Laber, 2015; Centanni et al., 2019; Sneddon et al., 2019; Yu et al., 2019; Flores-Bonilla and Richardson, 2020; M. A. Caruso et al., 2021; Rivera-Irizarry et al., 2023). However, with the advantages of the high temporal resolution of LIQ PARTI, we revealed that male mice display a unique pattern of ethanol consumption/preference. Male mice show a repetitive, undulating, within-week pattern of ethanol preference with the highest value occurring in concert with cage changes that diminishes over 24–48 h. While female mice have a persistently elevated ethanol preference each week, male mice begin each recording period with a preference for ethanol at similar levels to the female mice but dip to a very low preference by the end of the recording period. Given the length of time the male mice take to diminish their ethanol preference, the sensory cues for 10% ethanol, and the absence of this pattern in females, we believe this phenomenon is unlikely to be due simply to the swapping of bottle placement. This wave-like pattern of ethanol preference in males occurs immediately following cage changing and could represent a sensitivity to cage-change stress, as stress has been shown to increase ethanol consumption and preference in mice (Bahi, 2013; Anderson et al., 2016; M. J. Caruso et al., 2018; Reguilón et al., 2020). Alternatively, male C57Bl/6J mice have been shown to respond to novel objects more than females (Frick and Gresack, 2003), which could partially explain this effect. These issues will need to be further interrogated in future studies. Furthermore, bout microstructure changes in response to prolonged ethanol exposure are significantly different between male and female mice (Fig. 2). We previously have shown that female mice display a decrease in average bout size and bout duration at both the water and ethanol bottles during chronic ethanol exposure when singly housed with the LIQ HD system (Petersen et al., 2023). We observed this same pattern in group-housed female mice, but male mice showed the opposite effect, with an increase in bout size and duration at both bottles compared with the water-only week (Fig. 2*B*,*C*). We also showed previously in singly housed female mice that lick frequency increased and ILI decreased at the ethanol bottle compared with the water bottle during chronic ethanol exposure (Petersen et al., 2023). Interestingly, group-housed female mice displayed an increased frequency at the water bottle compared with males and compared with the water-only week and increased ILI at the ethanol bottle compared with the water bottle and compared with the water-only week. Males only showed a decrease in lick frequency at the water bottle compared with the water-only week (Fig. 2*E*,*F*). Together this suggests that social enrichment during the acquisition of ethanol preference and sex alters how bout microstructure is affected following chronic ethanol exposure.

Social isolation prior to ethanol exposure has been shown to significantly increase ethanol intake in mice (Lopez et al., 2011; Sanna et al., 2011; Butler et al., 2014a; Lopez and Laber, 2015; Cortés-Patiño et al., 2016; Panksepp et al., 2017; Evans et al., 2020; Cullins and Chester, 2024). Here, we show that social isolation in ethanol-experienced male and female mice increased ethanol preference, total fluid intake, and altered bout microstructure. It is important to note that LIQ PARTI and LIQ HD differ slightly in their physical structure, but both systems use identical sippers in the same vertical orientation, and the LIQ HD source code was modified to include the same lick detection optimization and bout logging method as LIQ PARTI. Furthermore, we validated that mice on average consume the same amount of liquid (∼1.4 µl) per lick in both systems (Extended Data Fig. 3-1). Both male and female mice significantly increased their total lick number (Figs. 3*F*, 5*E*), fluid consumption (Fig. 5*F*), and ethanol preference (Figs. 3*G*, 5*G*,*H*) during social isolation. Interestingly, male mice no longer displayed the undulating pattern of ethanol preference that corresponded to cage-changing days but instead showed persistently elevated preference throughout the recording periods similar to female mice (Fig. 3*C*). This may be due to the stress of social isolation overwhelming the stress or novelty induced by cage changes. Moreover, male mice have a more rigid social dominance hierarchy than females (Karamihalev et al., 2020), and group-housed males may experience additional stress during cage changes due to the re-establishment of the social dominance hierarchy (Hurst, 1993; Gray and Hurst, 1995; Lidster et al., 2019), which would not be a factor during social isolation. In addition to male mice displaying elevated ethanol preference during social isolation at comparable levels to females in the group-housed environment, bout microstructure of the male mice, specifically bout size and duration, during social isolation closely resembled the bout microstructure of the female mice (Fig. 4). Both male and female mice had an increased average lick duration at both bottles during social isolation, with a greater lick duration at the water bottle compared with the ethanol bottle. Interestingly, the lick frequency in female mice between the two bottles flipped during social isolation, with a greater lick frequency at the ethanol bottle compared with the water bottle, which recapitulates our previous study using LIQ HD in female mice (Petersen et al., 2023).

Several factors have been shown to contribute to changes in ethanol drinking microstructure. The literature suggests that bout size and lick frequency are directly related to the palatability of a drinking solution (Davis and Levine, 1977; Baird et al., 2005; Dwyer et al., 2011; Dwyer, 2012; Johnson, 2018). However, although bout size and duration during social isolation are greater at the ethanol bottle compared with the water bottle, our data suggest that a decreased bout size and duration overall are associated with a significantly elevated ethanol preference. In contrast, lick frequency in female mice is significantly increased by social isolation at the ethanol bottle. Additionally, changes in lick microstructure may represent different behavioral strategies for consumption. For example, broadly, the rate of food or fluid intake has been suggested as an indicator of motivation (Johnson, 2018; Radke et al., 2021). Behavioral strategies in the context of ethanol intake have been particularly characterized in the context of quinine-adulterated ethanol intake in rodents, a model of compulsive ethanol intake. For example, Darevsky et al. (2019) describe reduced variability in lick measures, interpreted as greater automatic responding. Finally, circulating ovarian hormones may also factor into drinking microstructure. While previous studies have shown no change in ethanol consumption across the estrous cycle in female rodents (Ford et al., 2002; Satta et al., 2018; De Oliveira Sergio et al., 2024; for a summary of studies see Radke et al., 2021), estrous phase can influence ethanol bout frequency and size (Ford et al., 2002), suggesting that sex differences in microstructure may be in part driven by hormonal factors. The estrous cycle was not tracked in the current study to avoid disruptions in continuous drinking behavior; however, this represents a future direction of interest. LIQ PARTI represents an ideal tool for use in studying the implications of these factors on group-housed ethanol drinking behavior.

As mentioned previously, prior experience of social isolation has significant and persistent impacts on ethanol preference in mice (Lopez et al., 2011; Sanna et al., 2011; Butler et al., 2014a; Lopez and Laber, 2015; Cortés-Patiño et al., 2016; Panksepp et al., 2017; Evans et al., 2020; Cullins and Chester, 2024). Holgate et al. (2017) found that male C57Bl/6J mice with previous ethanol exposure showed an increased preference for ethanol in a drinking in the dark (DID) task when moved from an enriched environment to social isolation but were unable to assess resocialization with two-bottle choice in the home cage due to aggressive behavior. Fortunately, we did not observe aggressive behavior following resocialization with the same cage mates. We found that fluid consumption, ethanol preference, and bout microstructure mostly returned to presocial isolation values when the mice were reintroduced to group housing with LIQ PARTI (Figs. 5–7; Extended Data Fig. 7-1). During the first group-housed week postsocial isolation, male mice had a persistently elevated average lick number per day and fluid consumption compared with presocial isolation but female mice returned to presocial isolation values (Figs. 5*E*,*F*, 6*F*). Average total lick number per day and total fluid consumption modestly decreased in females during the second week of postsocial isolation and males returned to presocial isolation levels. In contrast, ethanol preference in male mice quickly returned to presocial isolation levels with the undulating pattern by the first week, but females had a significantly reduced preference during the first week of postsocial isolation and returned to presocial isolation values by the second week (Figs. 5*G*,*H*, 6*C*,*G*). Furthermore, bout microstructure in both male and female mice reverted to presocial isolation value with some minor exceptions (Fig. 7; Extended Data Fig. 7-1). For example, male mice showed a decreased bout size and bout duration at the ethanol bottle, and female mice had a slightly increased lick duration at both bottles and decreased lick frequency at the water bottle postsocial isolation compared with presocial isolation, but to a much lower degree than during social isolation. Thus, social isolation-induced changes to ethanol drinking behavior and bout microstructure in ethanol-experienced mice are highly plastic, largely reverting to the presocial isolation baseline within 2 weeks.

Loneliness in humans has historically been associated with contributing to and maintaining alcohol use and AUD; however, while those with AUD have been found to have heightened feelings of loneliness, the relationship between social interaction and alcohol use is unclear (Åkerlind and Hörnquist, 1992; Mowbray et al., 2014). The COVID-19 pandemic and lockdown period was an era of intense social isolation for many individuals worldwide. While there was an increase in alcohol sales per capita in the United States during the pandemic, there was a significant decrease in the average drinks consumed per day with no difference in drinking days per month, binge drinking days per month, or maximum drinks per day (Castaldelli-Maia et al., 2021; Moskatel and Slusky, 2023). Furthermore, alcohol sales and consumption by all metrics decreased in the United States during periods with a high incidence of COVID-19 (Moskatel and Slusky, 2023). Studies in Brazil (Moura et al., 2023), Germany (Deeken et al., 2022), Australia (Bade et al., 2021), and others (Acuff et al., 2022) similarly found that alcohol consumption was in general significantly lower during lockdown periods. However, increased alcohol consumption was significantly associated with both particularly high and particularly low degrees of subjective loneliness (Bragard et al., 2022; Haucke et al., 2024). Additionally, online engagement in an AUD recovery service was significantly decreased, and alcohol withdrawal hospitalizations and alcohol-related deaths significantly increased in the United States during the COVID-19 pandemic (Colditz et al., 2021; Sharma et al., 2021; White et al., 2022). Alcohol use, AUD, social isolation, and loneliness thus represent a complex interaction that requires further investigation with significant public health implications.

Novel tools, such as LIQ PARTI and LIQ HD, afford several advantages over traditional methods that will aid in the investigation of how socialization and social isolation affect consummatory behaviors and the underlying physiology in rodent models. For example, future research can include studies on housing influences on the acquisition of ethanol drinking behavior, social transmission of drinking behavior, the impact of competition in the group-housed setting, and the effect of mouse strain on social drinking. Furthermore, the LIQ systems are well poised for the investigation of individual differences in drinking behavior and bout microstructure, as we have briefly highlighted (Extended Data Figs. 1-4, 2-1), such as individual variability in binge-like behavior and the influence of social hierarchy in group-housed drinking.

In conclusion, our study demonstrates the utility of LIQ PARTI and LIQ HD in elucidating sex differences and the impact of social isolation on ethanol consumption and bout microstructure in C57Bl/6J mice. We observed distinct patterns of ethanol preference between male and female mice, with males exhibiting an intriguing undulating response to cage changes. Moreover, chronic ethanol exposure induced significant alterations in bout microstructure, influenced by both sex and social environment. Social isolation in the ethanol-experienced mice led to marked increases in ethanol intake and preference in both sexes, underscoring the role of social context in modulating ethanol drinking behavior. However, the transition back to group housing resulted in the reversal of social isolation-induced changes to ethanol drinking behaviors, highlighting the plasticity of these responses. Overall, our findings contribute to a deeper understanding of the complex interplay between sex, social environments, and ethanol consumption, with future implications for understanding the physiological mechanisms underlying the relationship between loneliness and alcohol use.

## Synthesis

Reviewing Editor: Sam Golden, The University of Washington Decisions are customarily a result of the Reviewing Editor and the peer reviewers coming together and discussing their recommendations until a consensus is reached. When revisions are invited, a fact-based synthesis statement explaining their decision and outlining what is needed to prepare a revision will be listed below. The following reviewer(s) agreed to reveal their identity: Melissa Herman, Elizabeth Glover.

The study by Petersen et al. introduces LIQ PARTI, an open-source system for monitoring voluntary ethanol consumption in group-housed mice using RFID tags. This system allows for the continuous tracking of drinking behaviors, revealing that male and female mice exhibit different ethanol preferences and drinking patterns, which change based on social isolation or group housing. Females showed a higher preference for ethanol than males, and both sexes increased consumption in isolation. The study underscores the importance of social setting on drinking behavior and sex differences, providing a valuable tool for research while suggesting some methodological clarifications. Reviewers appreciate the design but urge the authors to provide data on individual drinking patterns in group settings, clarify the influence of cage changes, and improve statistical presentation and interpretation. Additionally, the potential impacts of diet, enrichment, competition for spouts, and variability in individual drinking behavior need further discussion. Overall, the reviewers find the study promising but recommend addressing specific methodological and data presentation concerns to enhance its utility and clarity.

Overall the reviewers are enthusiastic, but have requested that the manuscript be revised and re-reviewed after addressing the following comments.

Reviewer 1:

The current study by Petersen et al., examined the impact of social setting in voluntary ethanol consumption using a novel mouse home cage lickometer system and radio frequency identifier (RFID) tags that the authors have named LIQ PARTI (Lick Instance Quantifier with Poly-Animal RFID Tracking Integration). LIQ PARTI is an open-source design that is compatible with standard vivarium home cages and tracks undisturbed licks and bout microstructure over prolonged drinking periods. Using LIQ PARTI and their previous system, LIQ HD, the authors compared drinking structure in group-housed and single housed male and female mice. The authors report that males and females have different drinking microstructure and ethanol preference in group housed settings, that both male and female mice increase drinking behavior in isolation, but females have a higher preference for 10% ethanol as compared to males in both housing conditions. Interestingly, drinking microstructure was plastic and changes in drinking following social isolation were largely reversed following re-housing. Overall, this is a well-designed and well-written study detailing the construction, validation, and use of an automated homecage drinking system that allows for social housing. As current rodent drinking models typically require isolated housing or expensive caging options, this represents a new and valuable tool for the field. This work has significant implications as housing condition is an important component of drinking behavior and (as the authors show), underscores key sex differences in ethanol drinking and preference in different housing conditions. There are only minor issues with clarification of methodology and communication of the findings that should be addressed (see specific comments below):

1. The authors are commended for their rigorous reporting of experimental design and measures of drinking microstructure. However, they should also report diet and enrichment conditions in their Methods, as both have been shown to impact drinking.

2. The authors do an impressive job of illustrating the components of the LIQ PARTI system, however it might be helpful to also include an illustration of the cages with placement of LIQ PARTI system, particularly in relation to food source or enrichment components (if applicable).

3. Did the authors observe individual variability in drinking behavior (in either group-housed or socially-isolated conditions)? How did the individual intake compare to with and between cages? Was there a relationship between individual intake and cage-wide drinking behavior (i.e. did cagemates drinking equivalent volumes or was there greater/lesser intake by individual subject? Were there greater or lesser drinking cages)?

4. The fluctuating change in preference in males that coincides with experimental changes (increasing EtOH dose and/or different housing conditions) could reflect greater effects of novelty in addition to cage disruption stress. The authors may wish to expand on this point in their discussion.

5. Did the authors observe any evidence of competition for the drinking spouts in the group housed condition? As access to spouts is only granted to single subjects, it is possible that some subjects were forced to wait for access which may have contributed to decreased drinking behavior is group-housed as compared to isolated conditions. The authors may want to see if they have any evidence of competition (i.e. mice waiting for other mice to finish- would be particularly in the hypertonic saline injected group) or add text considering the potential impact of competition.

6. The authors may want to add text considering how social drinking may be different in different strains? There is no need to provide additional data for this point, just additional discussion of this important topic.

7. The authors report that all studies measuring fluid intake were conducted during the light cycle. As drinking behavior can vary by light cycle (and many drinking studies are conducted during the dark cycle), the authors may wish to speculate on how his may have impacted their findings.

8. The experimental design is well-suited to investigate housing-specific effects on drinking behavior, however the use of escalating concentrations in the group housed condition that occurs prior to social isolation and re-housing precludes a direct examination of the differences in drinking behavior over time between housing conditions. Particularly as ethanol drinking behavior has been reported to involve time-dependent increases in intake, the authors may wish to consider (speculate on) how housing condition impacts early drinking experience as well as changes over time (and concentration). Authors could also identify this as a focus of future studies.

Minor:

-In Figure 5, the y-axis labels for 5A and 5B use different water designations ("Water" vs H2O). This is not particularly confusing as both terms are common, but the authors may wish to standardize for consistency.

Reviewer 2:

The authors describe LIQ PARTI - a newly developed adaptation of their previously published lickometer-equipped home cage drinking device, LIQ HD, that allows for precise measurement of drinking structure in group housed mice. Historically, measurement of fluid intake in group housed rodents has been difficult and limited in large part by the exorbitant cost of sophisticated equipment. LIQ PARTI provides researchers with a low-cost alternative that is likely to propel forward the field's ability to explore social impacts on alcohol consumption. The authors do a nice job of providing build instructions and illustrating the kind of data that can be acquired using the device. Unfortunately, the authors declined to show data following individual intake within the group setting, which represents a significant missed opportunity that would arguably highlight a fundamental strength of LIQ PARTI. Minor concerns regarding data presentation and overstated conclusions also require resolution before this manuscript is suitable for publication.

Major:

1. The authors convey in the methods that LIQ PARTI is designed to track individual fluid intake in a group setting via RFID reader. The supplementary video provided shows this. However, the authors do not provide any data in the manuscript that demonstrates this ability! This is a significant missed opportunity and arguably the main advantage of this system. As it stands now, the same data from group housed animals shown in all figures of the manuscript could be acquired with any lickometer-equipped system (without RFID or other mechanism for individual identification). There are many, many questions the authors can investigate with the existing data set by examining individual behavior. In my opinion, this is of greater importance than providing an extensive comparison of group vs isolation drinking, some of which could be relocated to the supplement (e.g., Figs 5 &6 are largely redundant). I am not suggesting that the authors provide an exhaustive analysis but something demonstrating the unique advantage of LIQ PARTI over other less sophisticated systems would greatly improve the manuscript. Examples: Do individual animals show distinct patterns of intake that are consistent across days? Are there high/binge and low/sipping drinkers within a group? Alternatively, do animals within a cage drink similarly (but differences exist across cages)? Are high group drinkers also high drinkers during isolation? Do they increase drinking to the same degree as low group drinkers? Do high and low group drinkers return to same phenotype during re-socialization? Are there individual mice that change drinking strategy at one phase or another that is being obscured by looking at group averages?

2. The authors state that cage changes contributed to change in EtOH preference in males but it is unclear when changes occurred relative to change in behavior as depicted in Figure 1D. Is the first L-D cycle after each dotted line correspond to Mondays or some other day of the week? How can the authors be certain that cage changes are the driving force behind changes in behavior? If change in behavior occurs 24-48 hr after changes and changes occur on Fridays couldn't something over weekend correspond to change in behavior (e.g., male mice respond to quiet weekend hours in a way that facilitates drinking and this is diminished over the work week when more activity occurs in the facility)? The statements throughout re: cage changes and stress effects seem highly speculative to me. Without additional supporting data, I suggest tempering these statements.

3. Figures comparing lick microstructure lack graphs depicting lick number. They also do not show volume change (mL) per bottle. I realize this is provided in the preceding figures, but it is collapsed across bottles and therefore obscures some information. I think it would also be of benefit for the authors to provide an indication of g/kg ethanol intake in these comparisons. I realize during group housing this will be an estimate (which authors can make clear) but still would be helpful to understand how many licks equate to how much g/kg intake over the course of a day/session/phase of the experiment and what potential level of intoxication that may equate to.

4. The authors do not provide statistical results in body of manuscript for data presented in Figure 5. In addition, depicting main effects and interactions via asterisks in the graph corner is unconventional and potentially misleading. I originally thought this was a legend with number of asterisks indicative of comparison (e.g., data points with **** asterisks on graph where ****time is indicated = data point where effect of time was statistically significant from some other [unknown] time point). I recommend that authors describe results in the text or a table and indicate significance using more conventional methods (e.g., different symbols for different comparisons; significance indicator in figure legend for main effect of sex). It is also unclear what the results are when significance is indicated for an interaction (e.g., panel B) but no asterisks are shown on specific data points. Does this indicate the interaction achieved significance but posthoc comparisons did not or is it the case that comparisons were not performed? Are asterisks above data points indicative of significant interaction or of main effect of time relative to water or other time point?

Minor:

5. In the Intro, the authors describe "undisturbed licks" but it is unclear what is meant by this term

6. In the Methods, beginning on page 8, the authors describe modifications that need to be made to the Data Logger Shield. Are these modifications depicted in still photos or video associated with the previous publication or on the authors' github page? If yes, explicit statement of this would be helpful (e.g., following steps outlined in methods section of Petersen et al). If no, I recommend adding this as a figure or supplement to the current manuscript. It is possible that the statement at the end of this section on page 9 is serving this purpose but it would be beneficial to say this at top of section instead of the end.

7. There are several instances where statements in the Results are redundant with what has already been provided in the Methods (e.g., page 14 first paragraph, page 15)

8. Please provide an explicit statement in the Results when discussing Fig 2 that comparison is being made between the H2O week and the 4th week of 10% EtOH prior to single housing

9. Typo page 19 second sentence, last paragraph

10. I am unclear on what is happening when the authors say mice were group housed for two recording periods each 8 days long. Were these 8 day periods separated by something or was this 16 consecutive days? What happened between these two 8 day periods?

11. It is unclear if the number of sessions used for data depicted in Figures 1, 3 is the same data as used for Figures 2, 4.

12. Suppl Fig 2F-M - please clarify in Results and figure caption whether these data reflect water only sessions, water only bottles, or water and ethanol. Also, it would be helpful in the figure caption to convey that these measures are 7d measures (e.g., 80,000 licks per week), which I assume is the case.

## Author Response

Dear Dr. Golden, Here we resubmit our manuscript eN-TNWR-0234-24R1 entitled "A novel mouse home cage lickometer system reveals sex- and housing-based influences on alcohol drinking" for consideration for publication in eNeuro. We sincerely thank the reviewers for their insightful comments and constructive suggestions, which have greatly contributed to the improvement of our manuscript. We also extend our gratitude to the editor for the opportunity to revise and resubmit our work. We have carefully addressed all the points raised and provided detailed responses to each comment. Changes in the manuscript appear in blue text.

Manuscript Instructions 1. Extended Data should be labeled as Figure 1-1, Figure 1-2, Table 1-1, etc., so they indicate which figure they are supporting (i.e. Extended Data table supporting Figure 5 labeled as Figure 5-1). This change is not necessary at this stage, but will be if your paper receives a revise decision.

The extended data labels and the corresponding captions have been changed.

--------------------------------------------- Synthesis of Reviews:

Synthesis Statement for Author (Required):

The study by Petersen et al. introduces LIQ PARTI, an open-source system for monitoring voluntary ethanol consumption in group-housed mice using RFID tags. This system allows for the continuous tracking of drinking behaviors, revealing that male and female mice exhibit different ethanol preferences and drinking patterns, which change based on social isolation or group housing. Females showed a higher preference for ethanol than males, and both sexes increased consumption in isolation. The study underscores the importance of social settings on drinking behavior and sex differences, providing a valuable tool for research while suggesting some methodological clarifications. Reviewers appreciate the design but urge the authors to provide data on individual drinking patterns in group settings, clarify the influence of cage changes, and improve statistical presentation and interpretation. Additionally, the potential impacts of diet, enrichment, competition for spouts, and variability in individual drinking behavior need further discussion. Overall, the reviewers find the study promising but recommend addressing specific methodological and data presentation concerns to enhance its utility and clarity.

Overall the reviewers are enthusiastic but have requested that the manuscript be revised and re-reviewed after addressing the following comments.

Reviewer 1:

The current study by Petersen et al., examined the impact of social setting in voluntary ethanol consumption using a novel mouse home cage lickometer system and radio frequency identifier (RFID) tags that the authors have named LIQ PARTI (Lick Instance Quantifier with Poly-Animal RFID Tracking Integration). LIQ PARTI is an open-source design that is compatible with standard vivarium home cages and tracks undisturbed licks and bout microstructure over prolonged drinking periods. Using LIQ PARTI and their previous system, LIQ HD, the authors compared drinking structure in group-housed and single housed male and female mice. The authors report that males and females have different drinking microstructure and ethanol preferences in group housed settings, that both male and female mice increase drinking behavior in isolation, but females have a higher preference for 10% ethanol as compared to males in both housing conditions. Interestingly, drinking microstructure was plastic and changes in drinking following social isolation were largely reversed following re-housing. Overall, this is a well-designed and well-written study detailing the construction, validation, and use of an automated homecage drinking system that allows for social housing. As current rodent drinking models typically require isolated housing or expensive caging options, this represents a new and valuable tool for the field. This work has significant implications as housing condition is an important component of drinking behavior and (as the authors show), underscores key sex differences in ethanol drinking and preference in different housing conditions. There are only minor issues with clarification of methodology and communication of the findings that should be addressed (see specific comments below):

1. The authors are commended for their rigorous reporting of experimental design and measures of drinking microstructure. However, they should also report diet and enrichment conditions in their Methods, as both have been shown to impact drinking.

We have clarified the diet and enrichment conditions in the Methods section. Mice were given LabDiet 5LOD and Diamond Soft paper bedding with no additional enrichment beyond exposure to the LIQ devices.

2. The authors do an impressive job of illustrating the components of the LIQ PARTI system, however it might be helpful to also include an illustration of the cages with placement of LIQ PARTI system, particularly in relation to food source or enrichment components (if applicable).

We have included detailed instructions on the LIQ PARTI GitHub page (https://github.com/nickpetersen93/LIQ_PARTI) and a step-by-step tutorial video on YouTube (https://www.youtube.com/watch?v=k1wk4YVQ9fc), which includes footage of the device in a cage and an illustration of the cage on a ventilated rack at timestamps 12:05 and 13:08, respectively. We have included the link to the GitHub page at the beginning of the "LIQ PARTI Build" section in the methods for better visibility.

3. Did the authors observe individual variability in drinking behavior (in either group-housed or socially-isolated conditions)? How did the individual intake compare with and between cages? Was there a relationship between individual intake and cage-wide drinking behavior (i.e. did cagemates drinking equivalent volumes or was there greater/lesser intake by individual subject? Were there greater or lesser drinking cages)? We agree with this reviewer and believe the ability to capture individual variability is a huge advantage of the LIQ PARTI system. Accordingly, we have included extended figures highlighting individual variability of bout microstructure across animals in the LIQ PARTI system (during water and ethanol). Given the amount of additional data and the detailed analyses required to do the due diligence of properly exhibiting the individual variability data, these further analyses are not included in the current manuscript. We have also included a discussion point emphasizing these systems' potential advantages in studying individual variability in drinking behavior.

Please see Reviewer 2's first point as well.

4. The fluctuating change in preference in males that coincides with experimental changes (increasing EtOH dose and/or different housing conditions) could reflect greater effects of novelty in addition to cage disruption stress. The authors may wish to expand on this point in their discussion.

We have clarified in the Methods and the figures when cage changes occur and expanded on the discussion regarding stress and novelty.

5. Did the authors observe any evidence of competition for the drinking spouts in the group housed condition? As access to spouts is only granted to single subjects, it is possible that some subjects were forced to wait for access which may have contributed to decreased drinking behavior is group-housed as compared to isolated conditions. The authors may want to see if they have any evidence of competition (i.e. mice waiting for other mice to finish- would be particularly in the hypertonic saline injected group) or add text considering the potential impact of competition.

This is a very interesting point that we have not yet explored but believe is an important future direction. Given the short recording time and that only 2-3 animals per cage were injected with hypertonic saline during the validation video recordings, we did not observe any obvious competition at the sippers. With the advent of these devices and other open-source tools (such as SimBA for home cage video tracking of social behavior), we are excited to see what will be revealed in future work within this developing field. We have included a discussion point of the potential impact of competition in the group-housed setting.

6. The authors may want to add text considering how social drinking may be different in different strains. There is no need to provide additional data for this point, just additional discussion of this important topic.

We have added an additional discussion point on the effect of strain on social drinking.

7. The authors report that all studies measuring fluid intake were conducted during the light cycle. As drinking behavior can vary by light cycle (and many drinking studies are conducted during the dark cycle), the authors may wish to speculate on how this may have impacted their findings.

We apologize for this confusion. All drinking data collected by LIQ systems, both HD and PARTI, were collected continuously across 3-8 days of uninterrupted bottle access. At the end of recording periods, experimenters weighed bottles and changed cages during the light cycle to minimize disruption of drinking behavior since drinking is lowest during the light cycle. These weight values were then used to correlate LIQ system data with fluid intake across the entire recording period for validation purposes. Interruptions between recording periods lasted about 20 minutes to 1.5 hours, depending on the number of cages and experimenters. This has been clarified in the methods.

8. The experimental design is well-suited to investigate housing-specific effects on drinking behavior, however the use of escalating concentrations in the group housed condition that occurs prior to social isolation and re-housing precludes a direct examination of the differences in drinking behavior over time between housing conditions. Particularly as ethanol drinking behavior has been reported to involve time-dependent increases in intake, the authors may wish to consider (speculate on) how housing condition impacts early drinking experience as well as changes over time (and concentration). Authors could also identify this as a focus of future studies.

We completely agree and believe that this technology will open the door for future studies to explore the nuanced effects of housing on drinking behavior. We have expanded the discussion to speculate on this, such as the potential housing influences on the acquisition of drinking behavior and social transmission of behavior.

Minor:

1. In Figure 5, the y-axis labels for 5A and 5B use different water designations ("Water" vs H2O). This is not particularly confusing as both terms are common, but the authors may wish to standardize for consistency.

We have updated the figures for consistency.

- Reviewer 2:

The authors describe LIQ PARTI - a newly developed adaptation of their previously published lickometer-equipped home cage drinking device, LIQ HD, that allows for precise measurement of drinking structure in group housed mice. Historically, measurement of fluid intake in group housed rodents has been difficult and limited in large part by the exorbitant cost of sophisticated equipment. LIQ PARTI provides researchers with a low-cost alternative that is likely to propel forward the field's ability to explore social impacts on alcohol consumption. The authors do a nice job of providing build instructions and illustrating the kind of data that can be acquired using the device. Unfortunately, the authors declined to show data following individual intake within the group setting, which represents a significant missed opportunity that would arguably highlight a fundamental strength of LIQ PARTI. Minor concerns regarding data presentation and overstated conclusions also require resolution before this manuscript is suitable for publication.

Major:

1. The authors convey in the methods that LIQ PARTI is designed to track individual fluid intake in a group setting via RFID reader. The supplementary video provided shows this. However, the authors do not provide any data in the manuscript that demonstrates this ability! This is a significant missed opportunity and arguably the main advantage of this system. As it stands now, the same data from group housed animals shown in all figures of the manuscript could be acquired with any lickometer-equipped system (without RFID or other mechanism for individual identification). There are many, many questions the authors can investigate with the existing data set by examining individual behavior. In my opinion, this is of greater importance than providing an extensive comparison of group vs isolation drinking, some of which could be relocated to the supplement (e.g., Figs 5 &6 are largely redundant). I am not suggesting that the authors provide an exhaustive analysis but something demonstrating the unique advantage of LIQ PARTI over other less sophisticated systems would greatly improve the manuscript. Examples: Do individual animals show distinct patterns of intake that are consistent across days? Are there high/binge and low/sipping drinkers within a group? Alternatively, do animals within a cage drink similarly (but differences exist across cages)? Are high group drinkers also high drinkers during isolation? Do they increase drinking to the same degree as low group drinkers? Do high and low group drinkers return to same phenotype during re-socialization? Are there individual mice that change drinking strategy at one phase or another that is being obscured by looking at group averages? We agree that a huge advantage of the LIQ PARTI system is the ability to record individual drinking behavior in social housing, and these are fantastic questions that we hope to answer in subsequent studies. To highlight the ability to identify individual variability, in the revised manuscript we have included extended figures showing differences in bout microstructure across animals in the LIQ PARTI (during water and ethanol). As mentioned above (see response to Review 1, comment #2), the LIQ systems generate a very large amount of data, such that a proper in-depth analysis of individual differences in drinking behavior is beyond the scope of this manuscript.

2. The authors state that cage changes contributed to change in EtOH preference in males but it is unclear when changes occurred relative to change in behavior as depicted in Figure 1D. Is the first L-D cycle after each dotted line correspond to Mondays or some other day of the week? How can the authors be certain that cage changes are the driving force behind changes in behavior? If change in behavior occurs 24-48 hr after changes and changes occur on Fridays couldn't something over weekend correspond to change in behavior (e.g., male mice respond to quiet weekend hours in a way that facilitates drinking and this is diminished over the work week when more activity occurs in the facility)? The statements throughout re: cage changes and stress effects seem highly speculative to me. Without additional supporting data, I suggest tempering these statements.

We apologize for this confusion. The vertical dotted lines in the time series data (depicted in Figures 1, 3, and 6, and Extended Figures 1-5 and 7-1) represent when recordings were briefly paused for experimenters to weigh bottles and change cages. As shown in the figures, changes in the ethanol preference behavior in male mice in LIQ PARTI directly aligned with cage changes, thus we are confident this change in behavior is tightly associated with this experimenter intervention. More specifically, cage change and bottle weight measurements were always taken at the end of a recording period. Cages were changed on Fridays (start of water week and 3% ethanol) and on Mondays (start of 7% ethanol and 10% ethanol weeks). The last cage change occurred on a Tuesday due to the 8-day-long recording period. The initial manuscript submission mistakenly stated all cage changes and bottle weighing occurred on Fridays, which has been corrected in the revised manuscript.

3. Figures comparing lick microstructure lack graphs depicting lick number. They also do not show volume change (mL) per bottle. I realize this is provided in the preceding figures, but it is collapsed across bottles and therefore obscures some information. I think it would also be of benefit for the authors to provide an indication of g/kg ethanol intake in these comparisons. I realize during group housing this will be an estimate (which authors can make clear) but still would be helpful to understand how many licks equate to how much g/kg intake over the course of a day/session/phase of the experiment and what potential level of intoxication that may equate to.

We would like to emphasize that while the LIQ systems are highly accurate in their lick detection, they are not able to measure fluid changes. Changes in bottle weights were only taken at the end of recording periods to reduce experimenter interruptions in continuous recordings. However, we can estimate the intake volume using the correlation data, which shows an average of 1.4µL per lick (Extended Figure 1-2F). We have added graphs with the average daily lick number separated by bottle for Figures 1, 3, and 6. The average daily volume change from each recording period at the water bottle, ethanol bottle, and both bottles combined is shown in Figure 5. Although an estimate, we have added the "Estimated µL/hour" to the right Y-axis of hourly lick graphs. We have also added an extended figure with the "Estimated EtOH Intake (g/kg/hour)" and "Estimated EtOH Intake (g/kg/day)" at the ethanol bottle throughout the entire experiment.

4. The authors do not provide statistical results in body of the manuscript for data presented in Figure 5. In addition, depicting main effects and interactions via asterisks in the graph corner is unconventional and potentially misleading. I originally thought this was a legend with number of asterisks indicative of comparison (e.g., data points with **** asterisks on graph where ****time is indicated = data point where effect of time was statistically significant from some other [unknown] time point). I recommend that authors describe results in the text or a table and indicate significance using more conventional methods (e.g., different symbols for different comparisons; significance indicator in figure legend for main effect of sex). It is also unclear what the results are when significance is indicated for an interaction (e.g., panel B) but no asterisks are shown on specific data points. Does this indicate the interaction achieved significance but posthoc comparisons did not or is it the case that comparisons were not performed? Are asterisks above data points indicative of significant interaction or of main effect of time relative to water or other time point? The results for Figure 5 are provided in the second to last paragraph of the Results section. The main effects and interactions were displayed in the corner of the figures for ease but have been moved to the figure legend for clarity. The volume data on the right side of the figure has an n=2 for both males and females during the LIQ PARTI periods because the data points were calculated as the daily cage average [(total volume/number of mice) / number of days], which significantly reduced the statistical power. All asterisks above data points represent significant post-hoc comparisons between sexes, thus data points without asterisks represent comparisons that did not reach significance. Post-hoc comparisons across time were not calculated for this figure. The text and figure legend has been expanded to clarify the statistical methods.

Minor:

5. In the Intro, the authors describe "undisturbed licks" but it is unclear what is meant by this term By "undisturbed licks" we mean that the animals are not disturbed by human experimenters during the recording periods, only during weekly bottle weighing and cage changing. We have clarified this in the intro.

6. In the Methods, beginning on page 8, the authors describe modifications that need to be made to the Data Logger Shield. Are these modifications depicted in still photos or video associated with the previous publication or on the authors' github page? If yes, explicit statement of this would be helpful (e.g., following steps outlined in methods section of Petersen et al). If no, I recommend adding this as a figure or supplement to the current manuscript. It is possible that the statement at the end of this section on page 9 is serving this purpose but it would be beneficial to say this at top of section instead of the end.

Yes, we have a detailed guide and video tutorial on our GitHub page that shows all the steps to build the system, including modifying the Data Logger Shield. We have included the additional reference to our previous paper and have also moved the link to the GitHub to the beginning of the section.

7. There are several instances where statements in the Results are redundant with what has already been provided in the Methods (e.g., page 14 first paragraph, page 15) We have edited the text in the Results section to be more concise and reduce method redundancy.

8. Please provide an explicit statement in the Results when discussing Fig 2 that comparison is being made between the H2O week and the 4th week of 10% EtOH prior to single housing.

A statement has been added to the Results stating that Figure 2 compares the water week to the 4th week of 10% ethanol before single housing.

9. Typo page 19 second sentence, last paragraph We have fixed the typo.

10. I am unclear on what is happening when the authors say mice were group housed for two recording periods each 8 days long. Were these 8 day periods separated by something or was this 16 consecutive days? What happened between these two 8 day periods? As described above (Reviewer #1, comment #7), all recording sessions were conducted continuously across 3-8 days of uninterrupted bottle access, with experimenters weighing bottles and changing cages between sessions. Recordings were resumed immediately. This has been clarified in the text.

11. It is unclear if the number of sessions used for data depicted in Figures 1, 3 is the same data as used for Figures 2, 4.

Data were recorded continuously over 82 days, as summarized in Figure 5 and Extended Figures 1-5 and 7-1. The vertical dotted lines in the time series data (depicted in Figures 1, 3, and 6, and Extended Figures 1-5 and 7-1) represent when recordings were briefly paused for experimenters to weigh bottles and change cages. For analysis, the data were broken down into critical time periods, thus they are the same data but used to assess differences across time/across the different critical periods. These points have been clarified in the text and figure legends.

12. Suppl Fig 2F-M - please clarify in Results and figure caption whether these data reflect water only sessions, water only bottles, or water and ethanol. Also, it would be helpful in the figure caption to convey that these measures are 7d measures (e.g., 80,000 licks per week), which I assume is the case.

Extended Figure 1-2F-M includes compiled data from all bottle measurements throughout the study from the LIQ PARTI system, irrespective of fluid type or ethanol exposure, for validation purposes. The measurements are taken from the 3-8 day recording periods and each data point represents the totals of each LIQ parameter (lick number, lick duration, bout number, and bout duration) at the bottle (the sum of licks from all 5 mice in the cage) versus the total volume change of that bottle. This has been clarified in the Results and figure caption.
