# Supplementary figures and images for: A Novel Mouse Home Cage Lickometer System Reveals Sex- and Housing-Based Influences on Alcohol Drinking
Source: eNeuro. 2024 Oct 4;11(10):ENEURO.0234-24.2024. doi: 10.1523/ENEURO.0234-24.2024 (PMC11498228; doi:10.1523/ENEURO.0234-24.2024)

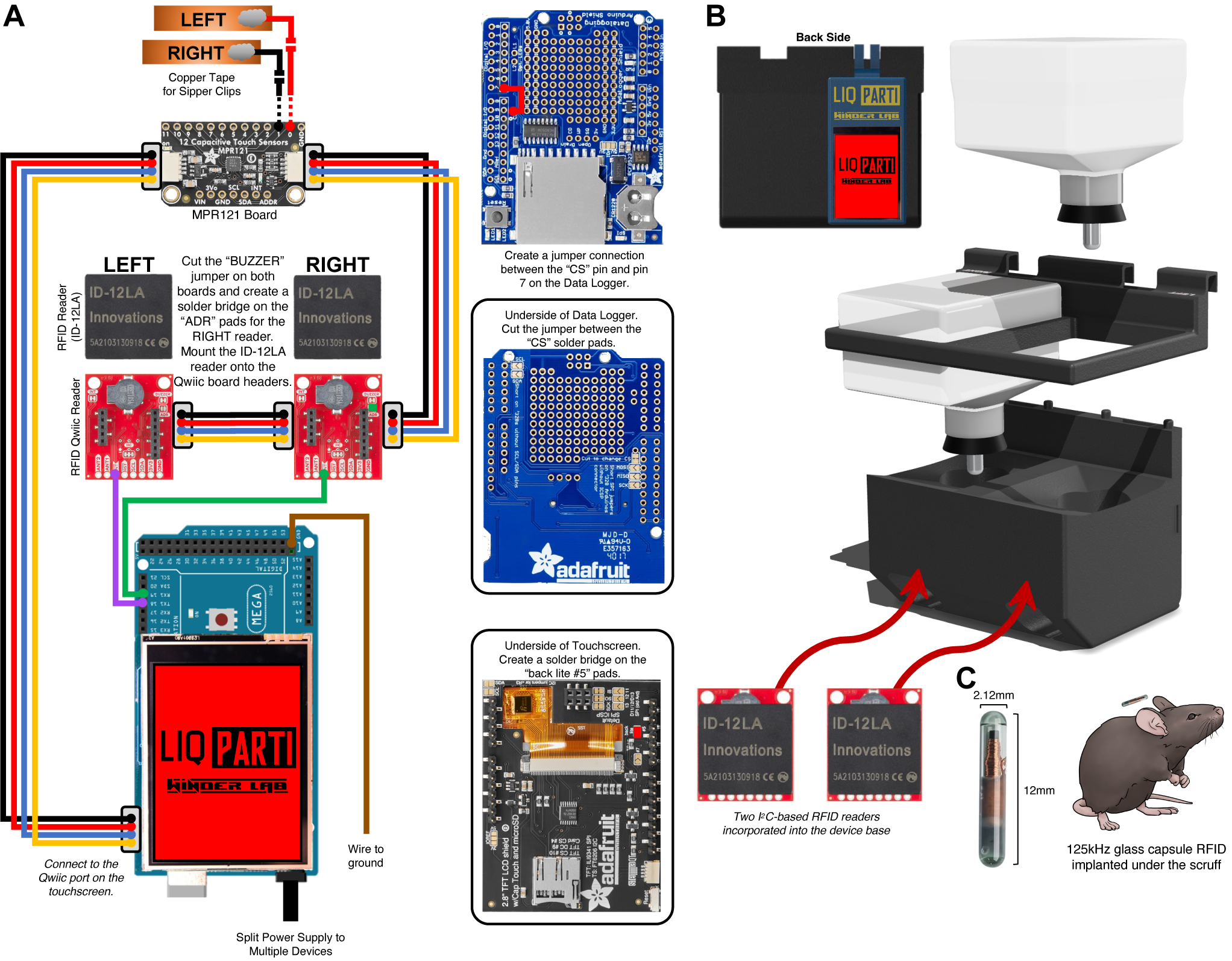

Supplement: Figure 1-1 — LIQ PARTI wiring diagram and build. A, LIQ PARTI electronic parts and wiring diagram including an Arduino Mega, two RFID readers, an MPR121 capacitive touch board, a touchscreen shield, a data logger shield, and conductive copper foil tape. B, 3D rendering of LIQ PARTI disassembled components, including 3D-printed parts, rubber stoppers with sippers, RFID readers, and Arduino microcontroller. C, Glass capsule RFID tag with dimensions and representative illustration of the RFID tag to scale (estimated) with a mouse. More detailed information can be found at (https://github.com/nickpetersen93/LIQ_PARTI). Download Figure 1-1, TIF file. [file eneuro-11-ENEURO.0234-24.2024-s004.tif]

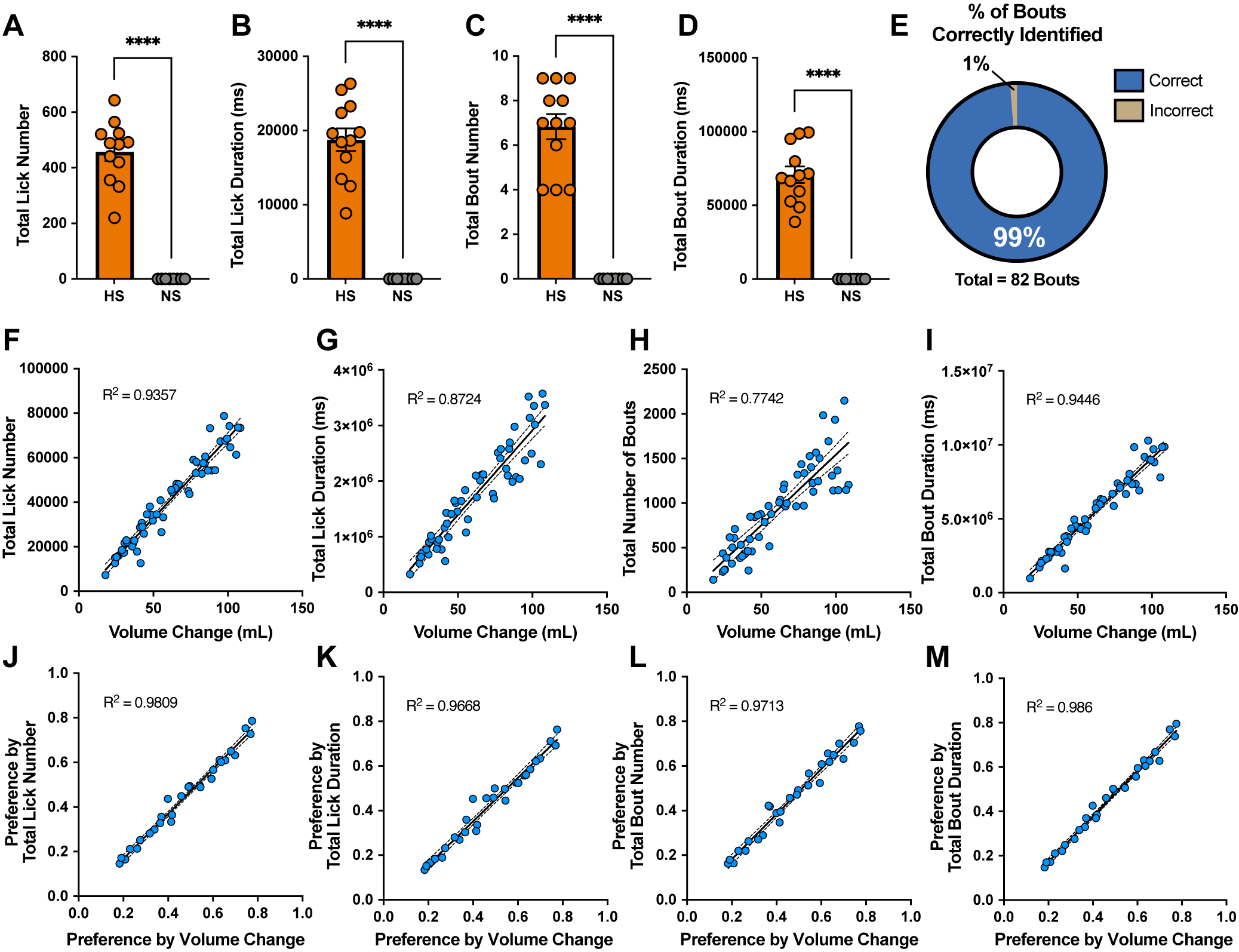

Supplement: Figure 1-2 — LIQ PARTI accurately identifies individual mice in a group-housed environment and lick and bout detection closely correlates with bottle weight measurements. Total lick number (A), lick duration (B), bout number (C), and bout duration (D) of mice injected with hypertonic saline (HS) or normal saline (NS) during the LIQ PARTI video recording of water drinking for the validation experiment (unpaired t-test; n = 12 mice HS group; n = 8 mice NS group). E, The percentage of video-recorded drinking bouts that were attributed by LIQ PARTI to the correct RFID-tagged mouse. Correlation between whole-cage total lick number and volume change (F), total lick duration and volume change (G), total bout number and volume change (H), and total bout duration and volume change (I) for each recording period. Correlation between preference calculated by total lick number and preference calculated by volume change (J), preference by total lick duration and preference by volume change (K), preference by total bout number and preference by volume change (L), and preference by total bout duration and preference by volume change (M) for each recording period. (F-M) includes compiled data from all LIQ PARTI bottle measurements, irrespective of fluid type or ethanol exposure, from the 3-8 day recording periods. In correlation graphs, solid lines represent a fitted simple linear regression model, and dashed lines denote 95% confidence intervals. Data in A-E are reported as individual mouse values and F-M are reported as whole-cage values. ****p < 0.0001. Download Figure 1-2, TIF file. [file eneuro-11-ENEURO.0234-24.2024-s005.tif]

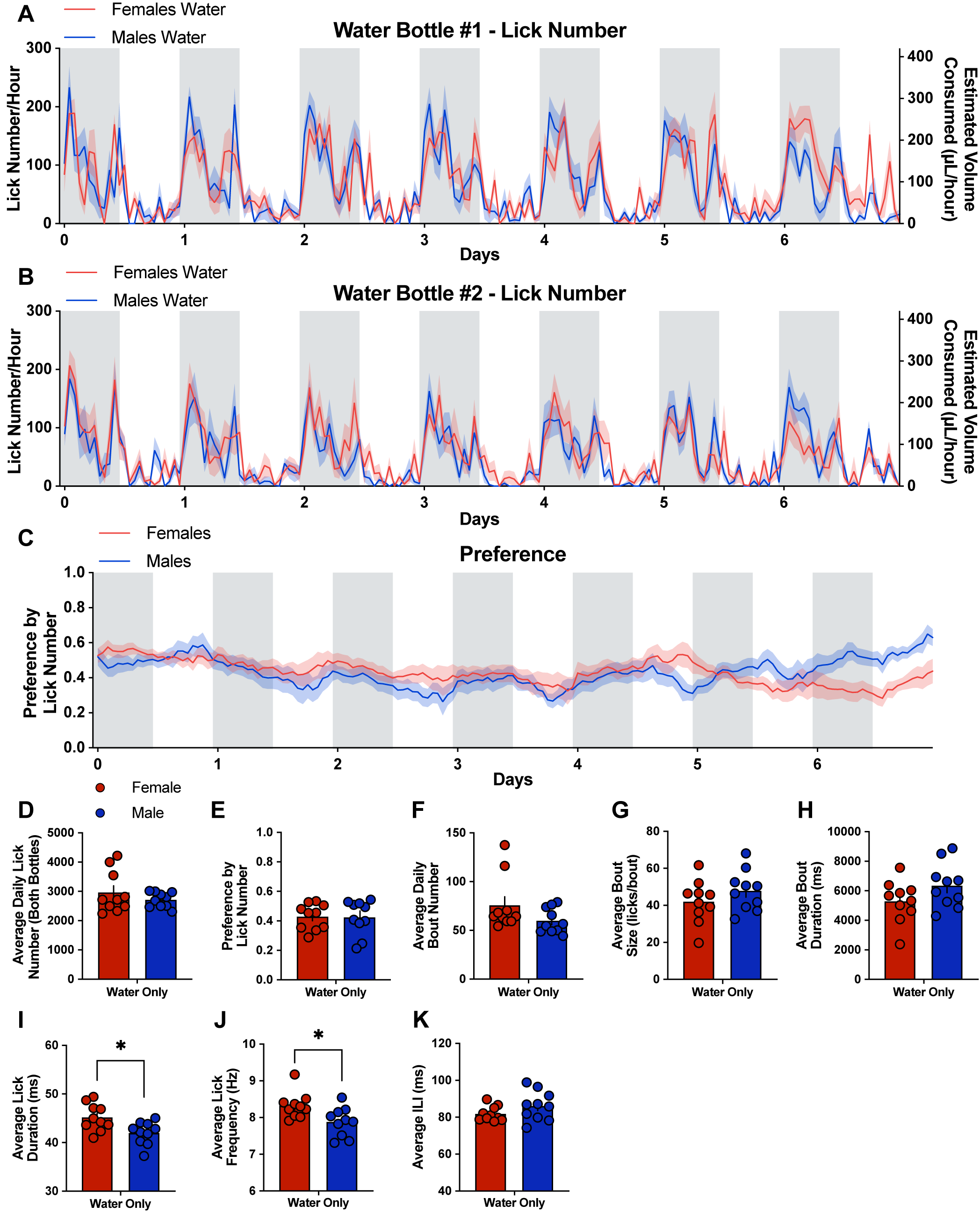

Supplement: Figure 1-3 — Group-housed male and female mice consume water at similar rates when given continuous access to two water bottles, and female mice show a significantly higher average lick frequency and lick duration compared to males at baseline. Lick number per hour and estimated volume consumed from male and female mice at the left water bottle (A) and the right water bottle (B) throughout the water-only week with the group-housed LIQ PARTI system. C, Bottle preference score calculated by lick number at each water bottle in male and female mice throughout the water-only week. The average lick number per day (D), average preference by lick number (E), average daily bout number (F), average bout size (G), average bout duration (H), average lick duration (I), average lick frequency (J), and average ILI (K) were calculated based on total values at both bottles and compared between males and females during the water-only week with LIQ PARTI (unpaired t-test). The solid lines (A-C) represent the mean lick number in 1-h bins, and the red/blue shaded areas represent ±SEM. Gray-shaded areas represent the dark cycle. Error bars (D-K) represent ±SEM. (n = 10 mice per group and reported as individual mice). *p < 0.05. Download Figure 1-3, TIF file. [file eneuro-11-ENEURO.0234-24.2024-s006.tif]

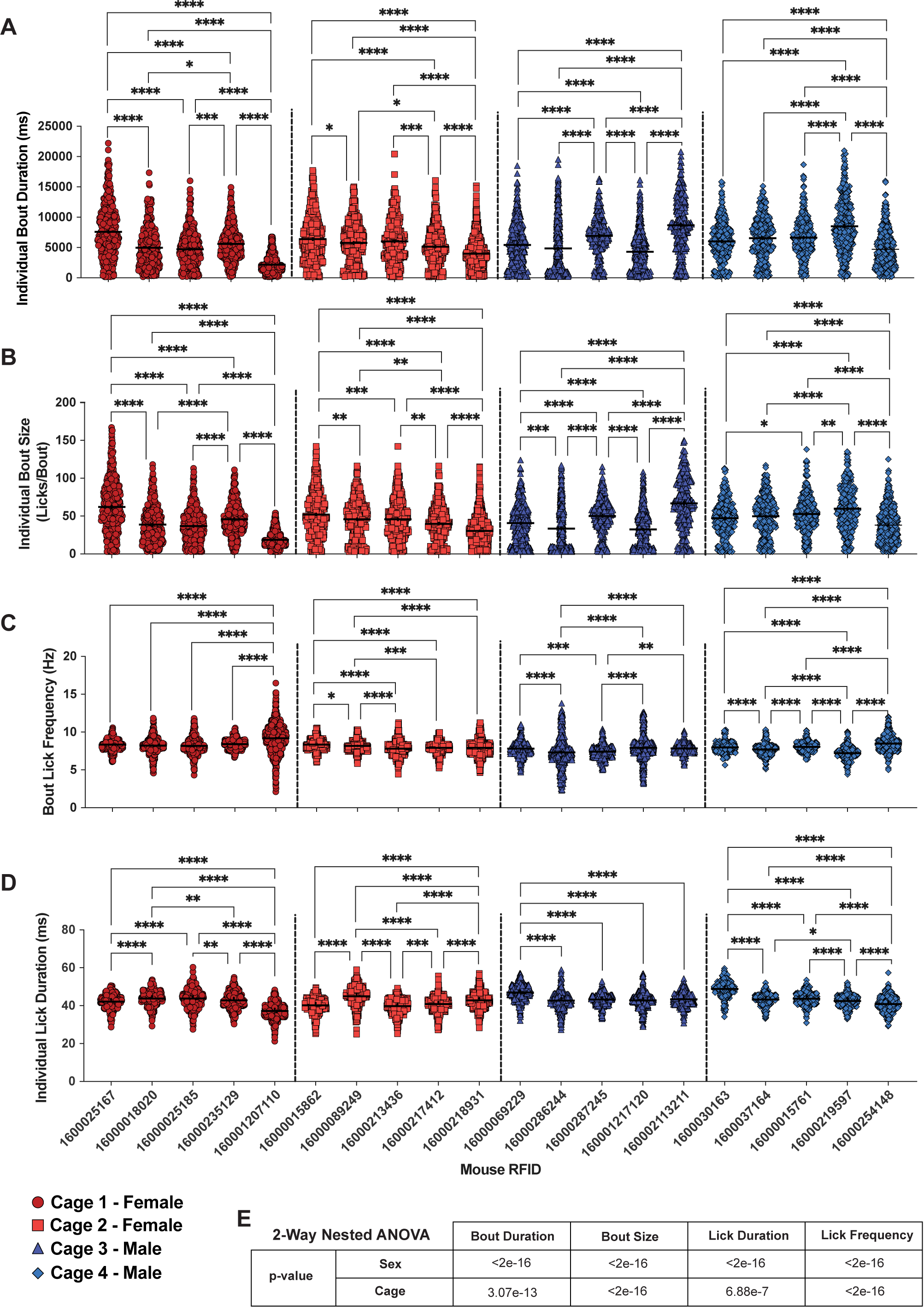

Supplement: Figure 1-4 — Bout microstructure is significantly different across sex, cage, and individual mice during the water-only week. Individual bout duration (A), individual bout size (B), bout lick frequency (C), and individual lick duration (D) for each bout during the water-only week (both water bottles combined). All bout microstructure parameters show significant differences between individual mice in the same cage (one-way ANOVA with Tukey’s test for multiple comparisons for within-cage comparisons). E, All bout microstructure parameters differ significantly across cages and between male and female mice (two-way nested ANOVA, main factor of sex, cage nested into sex). Each data point represents an individual bout that occurred during the water-only recording period. The number of bouts ranges from n = 302 to n = 934. The vertical dotted line separates mice in each cage. The solid horizontal line represents the sample means. Download Figure 1-4, TIF file. [file eneuro-11-ENEURO.0234-24.2024-s007.tif]

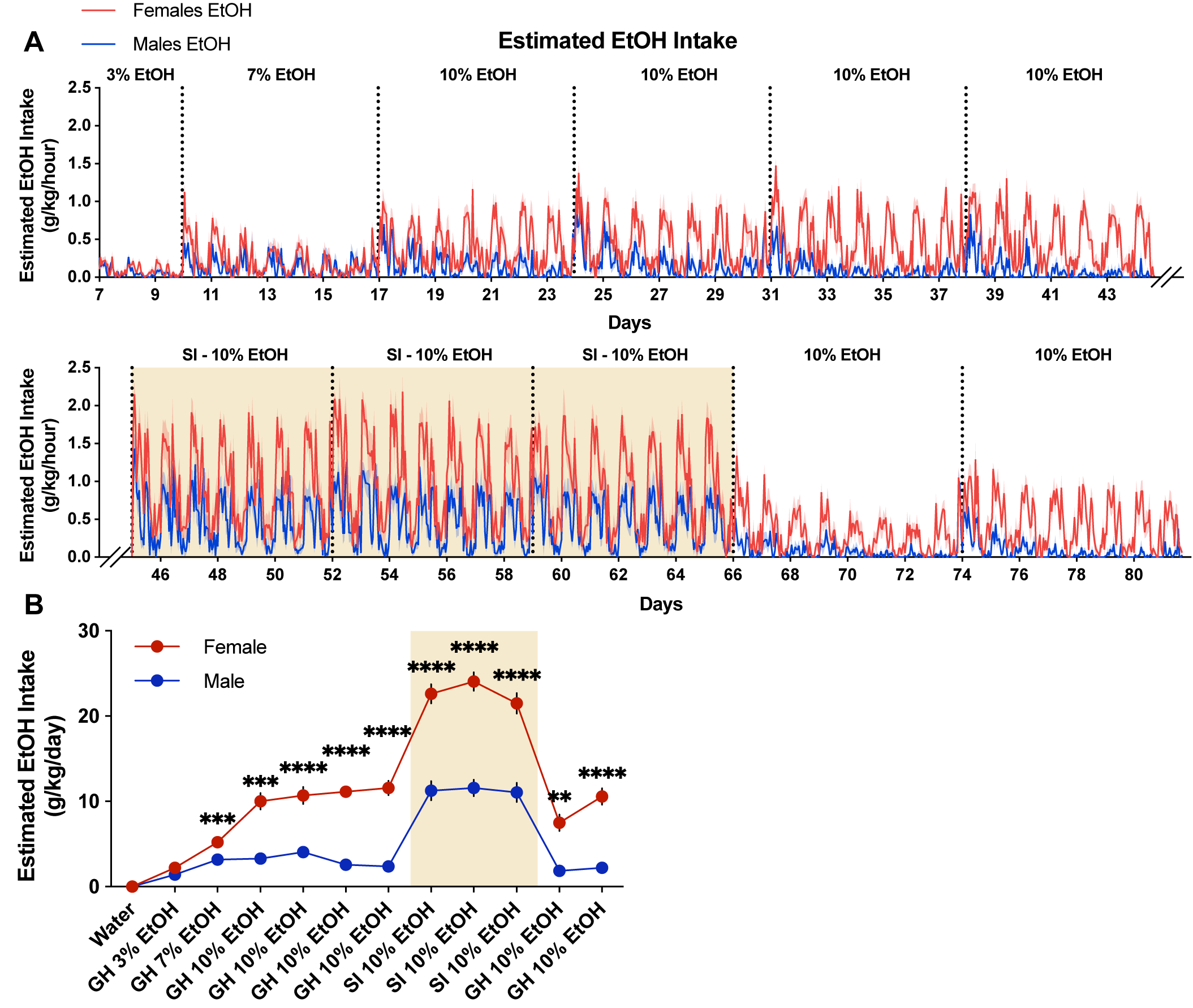

Supplement: Figure 1-5 — Estimated ethanol intake throughout the experiment. A, Estimated hourly ethanol intake over time across all ethanol-exposed recording periods. B, Estimated daily ethanol intake over time across all recording periods (repeated measures two-way ANOVA with the Geisser–Greenhouse correction and Šidák multiple comparisons test for female versus male at each time point; significant main effect of Time p<0.0001 and Sex p<0.0001, and significant interaction effect of Time x Sex p<0.0001; n = 10 mice per group). Yellow-shaded regions indicate periods of social isolation with LIQ HD. Error bars represent ±SEM. ***p < 0.001, ****p < 0.0001. Download Figure 1-5, TIF file. [file eneuro-11-ENEURO.0234-24.2024-s008.tif]

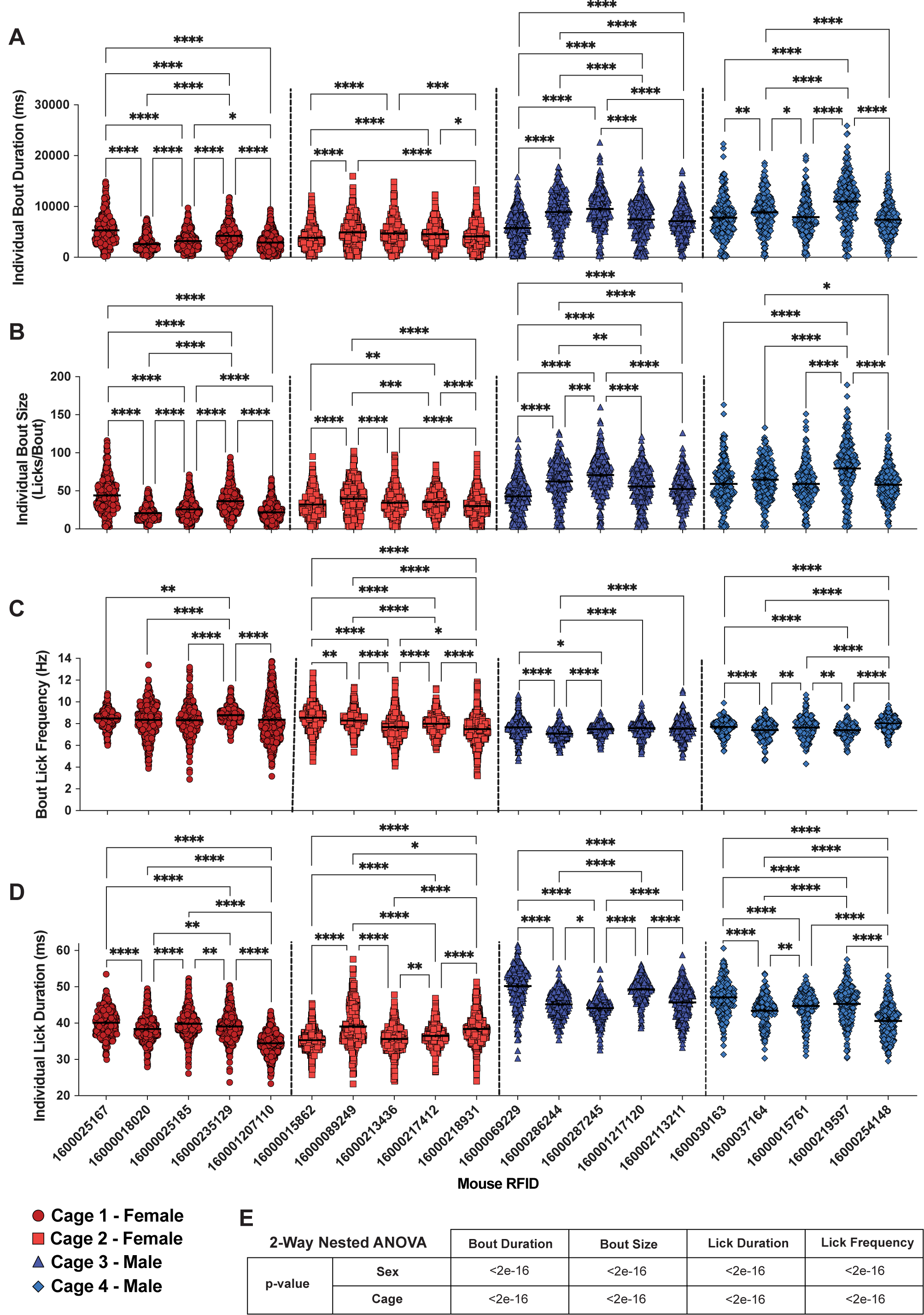

Supplement: Figure 2-1 — Bout microstructure is significantly different across sex, cage, and individual mice during the fourth week of 10% ethanol exposure. Individual bout duration (A), individual bout size (B), bout lick frequency (C), and individual lick duration (D) for each bout during the fourth week of 10% ethanol (both water bottles combined). All bout microstructure parameters show significant differences between individual mice in the same cage (one-way ANOVA with Tukey’s test for multiple comparisons for within cage comparisons). E, All bout microstructure parameters differ significantly across cages and between male and female mice (two-way nested ANOVA, main factor of sex, cage nested into sex). Each data point represents an individual bout that occurred during the fourth week of 10% ethanol exposure. The number of bouts ranges from n = 231 to n = 810. The vertical dotted line separates mice in each cage. The solid horizontal line represents the sample means. Download Figure 2-1, TIF file. [file eneuro-11-ENEURO.0234-24.2024-s009.tif]

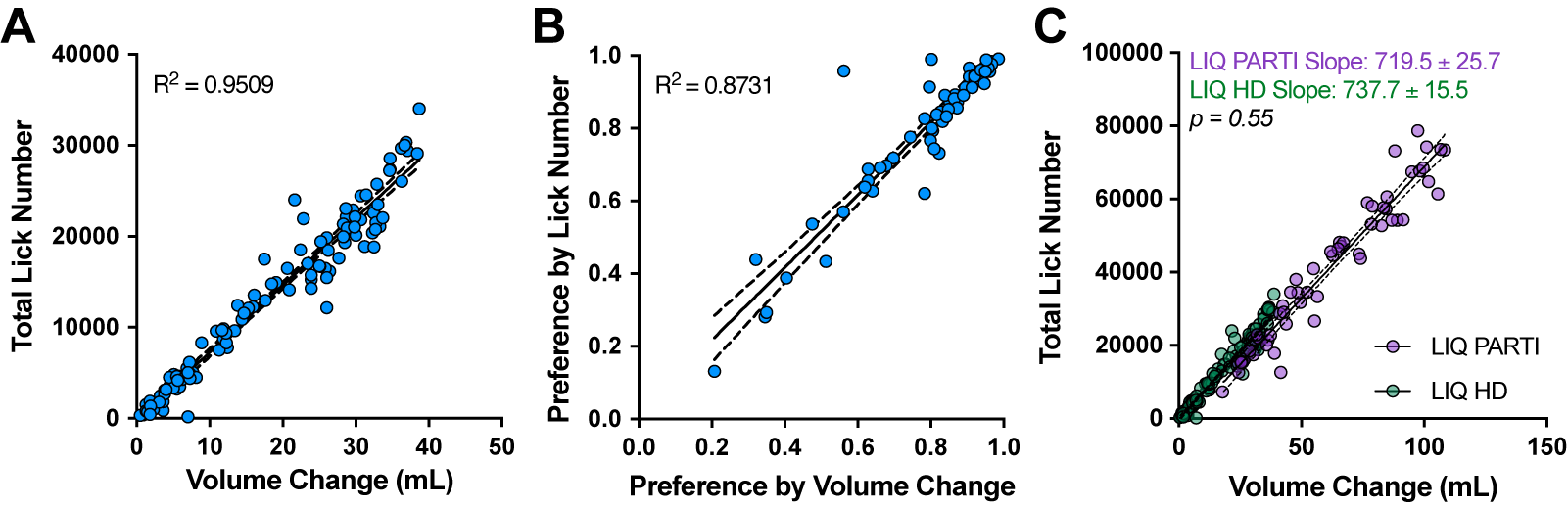

Supplement: Figure 3-1 — LIQ HD and LIQ HD vs LIQ PARTI validation. Correlation between total lick number and volume change (A) and correlation between preference calculated total lick number and preference by volume change (B) for each recording period of social isolation with LIQ HD. C, comparison of correlations between total lick number and volume change for LIQ HD and LIQ PARTI. There is no significant difference between the fitted region slopes for the LIQ HD and LIQ PARTI systems. Solid lines represent a fitted simple linear regression model, and dashed lines denote 95% confidence intervals. LIQ PARTI data are reported as whole-cage values. Download Figure 3-1, TIF file. [file eneuro-11-ENEURO.0234-24.2024-s010.tif]

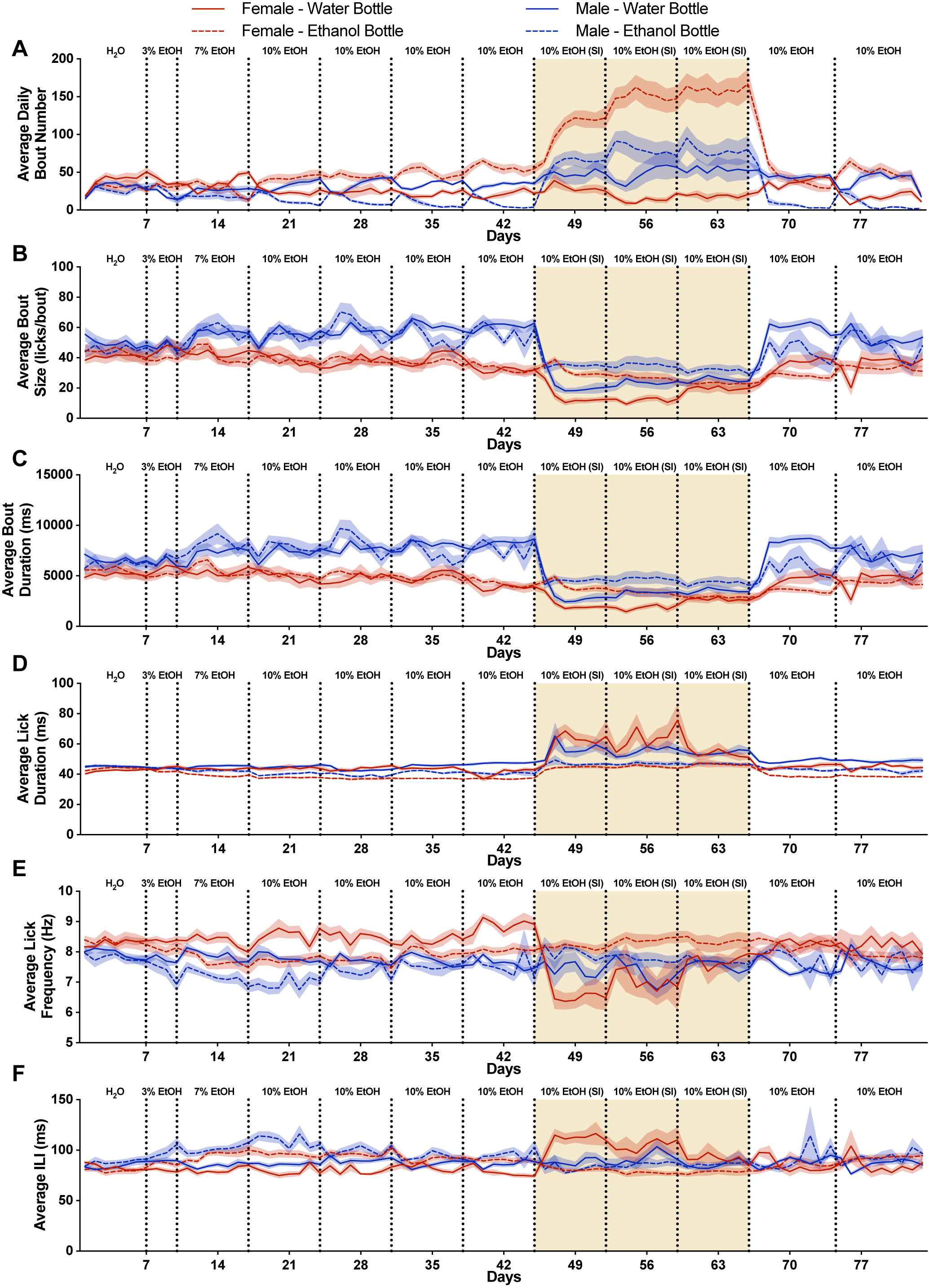

Supplement: Figure 7-1 — Exposure to ethanol and social isolation significantly alters drinking microstructure in a sex-, bottle, and housing-specific manner over time. Average daily bout number (A), bout size (B), bout duration (C), lick duration (D), lick frequency (E), and ILI (F) for male and female mice at the water and ethanol bottles. Lines represent the means in 24-h bins, and the red/blue shaded areas represent ±SEM. Yellow-shaded regions indicate periods of social isolation with LIQ HD and vertical dotted lines represent when cages were cleaned and bottle positions were swapped. (n = 10 mice per group, except males post-SI week 2 n = 5, and reported as individual mice). Download Figure 7-1, TIF file. [file eneuro-11-ENEURO.0234-24.2024-s011.tif]
